# Laboratory validation of targeted next-generation sequencing assay for pathogen detection in lower respiratory infection

**DOI:** 10.1128/spectrum.01751-24

**Published:** 2025-05-21

**Authors:** Jing Yang, Yan Wang, Li Yang, Jun Wu

**Affiliations:** 1 Beijing Jishuitan Hospital, Capital Medical University, Beijing, China; 2 Department of Laboratory Medicine, Beijing Jishuitan Hospital, Peking University Fourth School of Clinical Medicine, Beijing, China; 3 Chongqing School, University of Chinese Academy of Sciences, Chongqing, China; 4 Sansure Biotech Incorporation, Changsha, Hunan, China; FIND, Geneva, Switzerland

**Keywords:** tNGS, lower respiratory infection, laboratory validation, diagnosis, infection

## Abstract

Lower respiratory tract infection (LRTI) is a serious global public health issue and poses significant challenges for detection and diagnosis. We validated the detection performance of targeted next-generation sequencing (tNGS) based on multiplex PCR using simulated microbial sample panels and clinical samples, providing a theoretical basis for promoting and applying tNGS in clinical diagnosis. We used a series of simulated microbial sample panels to validate the analytical validity of tNGS comprehensively. We also use tNGS to test respiratory specimens from 108 diagnosed or suspected LRTI patients to validate its clinical validity in diagnosing LRTI. Finally, we summarize the drug-resistance genes obtained from tNGS, the detection cost, and the turnaround time. tNGS has good analytical specificity, sensitivity, and precision. It has good stability when stored under low-temperature conditions. Using the composite diagnostic criteria as the gold standard, our internal tNGS platform has a sensitivity of 84.38%, specificity of 91.67%, positive predictive value of 98.78%, and negative predictive value of 42.31%. In terms of turnaround time, tNGS (about 16 h) and metagenomic next-generation sequencing (about 24 h) are similar, both significantly superior to traditional microbial detection methods (3–5 days). The cost of tNGS is approximately one-fourth of metagenomic next-generation sequencing. As a novel method with acceptable performance and cost, tNGS can compensate for the shortcomings of commonly used pathogen detection schemes in clinical practice, and its application prospects are worth looking forward to.

**IMPORTANCE:**

Lower respiratory tract infection (LRTI) is a serious global public health problem, and detecting its pathogenic microorganisms is difficult. Targeted next-generation sequencing (tNGS) is a rising star in microbial detection, with enormous potential. To understand the detection performance of tNGS and provide a theoretical basis for promoting its application in clinical diagnosis, this study prepared simulated microbial sample panels using reference materials to evaluate the analytical and clinical validity of tNGS. Our research suggests that tNGS has good analytical specificity and sensitivity, precision, and stability. Additionally, it can reliably detect common LRTI pathogens. It has advantages in identifying co-infections and atypical pathogens. Moreover, tNGS significantly reduces turnaround time, allowing faster treatment. In summary, tNGS is expected to be used in clinical practice to diagnose and manage LRTI.

## INTRODUCTION

The high incidence and mortality of respiratory tract infections make them one of the most serious public health problems worldwide (1, 2). According to data from the World Health Organization (WHO) in 2019, lower respiratory tract infections (LRTIs) are the fourth leading cause of death globally and the deadliest infectious diseases (3). In contrast to other diseases, due to the diverse types, low concentrations, co-infection, and susceptibility to mutations, it is very difficult to identify the pathogens that cause LRTIs. Early, rapid, and accurate identification of pathogens is a prerequisite for effective diagnosis and treatment of LRTIs. However, a study based on large-scale population monitoring reported that only 38% of adults with community-acquired pneumonia were detected as pathogens (4).

Culture is a traditional method to identify pathogens and is the gold standard for the diagnosis of infectious diseases (5). However, this method has a relatively low sensitivity and a longer culture period. Quantitative polymerase chain reaction (PCR) is simple to perform, cost-effective, and widely used, but only for specific gene fragment detection. Metagenomic next-generation sequencing (mNGS), a non-targeted and unbiased detection method, can sequence all nucleic acid fragments in one detection process (6). It has great advantages for unknown pathogens and pathogens that cannot be routinely detected in most clinical microbiology laboratories because of the high culture requirements and long culture times. However, its operational steps are cumbersome, interpretation is complex, and the cost is high. Targeted next-generation sequencing (tNGS) combines multiplex PCR with sequencing to simultaneously detect hundreds of pathogens as well as virulence and drug-resistance genes. In July 2023, the WHO released the “use of targeted next-generation sequencing for diagnosis of drug-resistant tuberculosis” (7). Although this technology is still in the early stages of global application, it has received attention from the WHO, demonstrating its enormous diagnostic potential and value. But there are still many challenges in applying tNGS detection in clinical microbiology laboratories. It must be addressed through complete and comprehensive performance validation. However, there is currently a lack of research on this topic.

**Fig 1:**
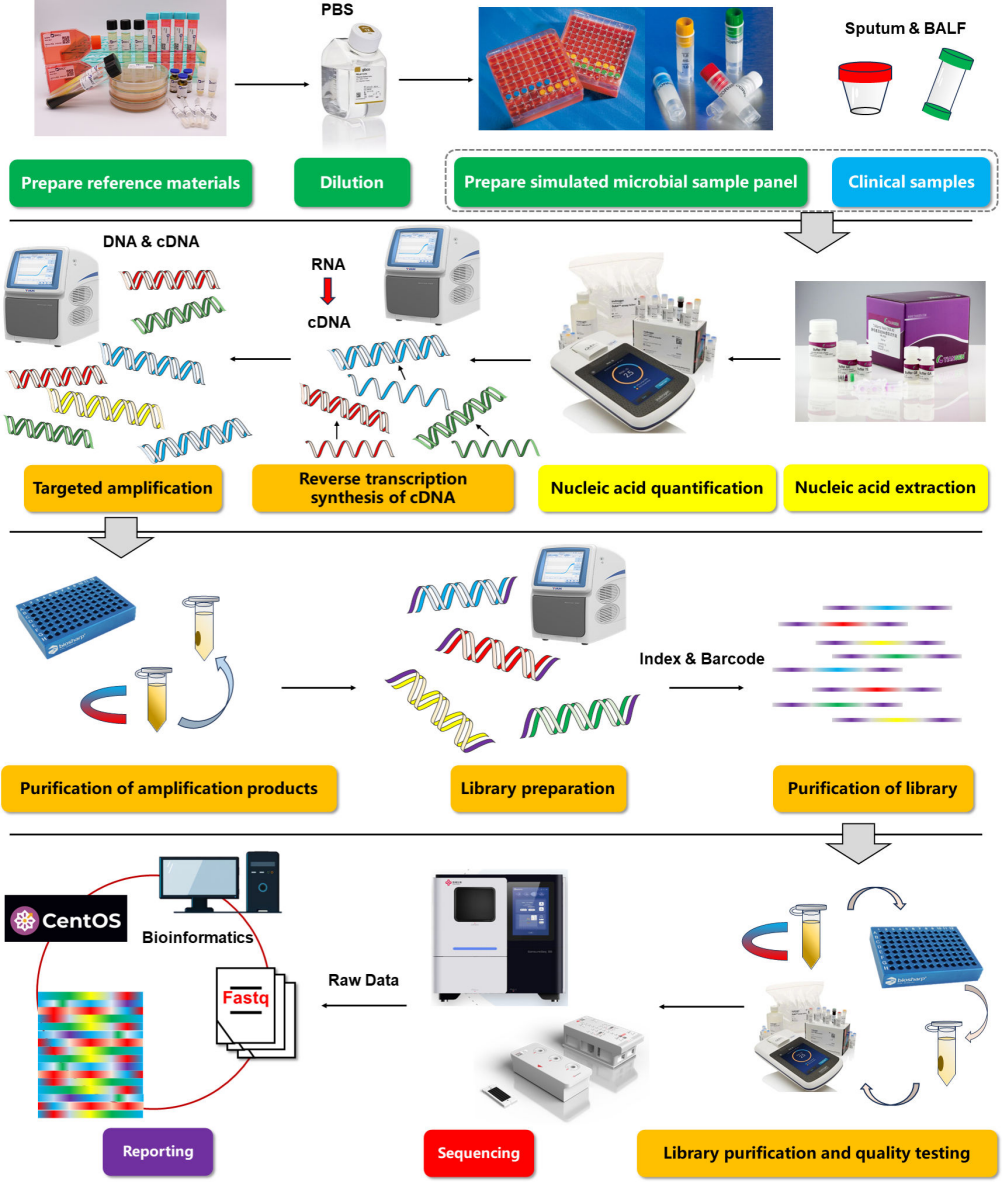
The validation performance workflow of in-house tNGS assay.

Therefore, the first part of this study designed simulated microbial sample panels containing different types and concentrations of common pathogens that cause LRTIs to verify the performance indicators and objectively evaluate the analytical validity of tNGS in the diagnosis of LRTIs (Fig. 1). In the second part of this study, tNGS, culture, and thermostatic amplification gene chip methods were used to detect sputum and bronchoalveolar lavage fluid (BALF) samples from patients, providing reliable evidence for their future clinical application. Finally, we summarize the turnaround time (TAT) of samples and drug-resistance gene information throughout the tNGS detection process to provide a simple evaluation of clinical utility. This study evaluated whether tNGS has good performance in the detection of common pathogens in LRTIs and established a method for detecting respiratory pathogens and diagnosing LRTIs based on tNGS in our laboratory. This study also provides a theoretical basis for the future application of tNGS in the diagnosis of clinical LRTIs.

## MATERIALS AND METHODS

### Patients and samples

Sputum or BALF samples were collected from inpatients or outpatients at Capital Medical University Affiliated Beijing Jishuitan Hospital. All of them have completed culture or thermostatic amplification gene chip tests to confirm the presence or absence of pathogens causing LRTIs. Among them, the thermostatic amplification gene chip is produced by CapitalBio Technology, and the detection principle is loop-mediated isothermal amplification. It can detect eight pathogens, including *Streptococcus pneumoniae*, *Staphylococcus aureus*, methicillin-resistant *Staphylococcus aureus*, *Klebsiella pneumoniae*, *Pseudomonas aeruginosa*, *Acinetobacter baumannii*, *Stenotrophomonas maltophilia*, and *Haemophilus influenzae*. The inclusion criteria are as follows: (i) patients clinically diagnosed with LRTIs; (ii) patients with symptoms such as acute cough, expectoration or chronic cough exacerbation, cough white or yellow mucopurulent sputum, etc., supported by clear chest imaging and laboratory examination data; (iii) patients who have completed cultivation or gene chip tests for pathogen detection.

### Evaluation of tNGS analytical performance characteristics

**Fig 2:**
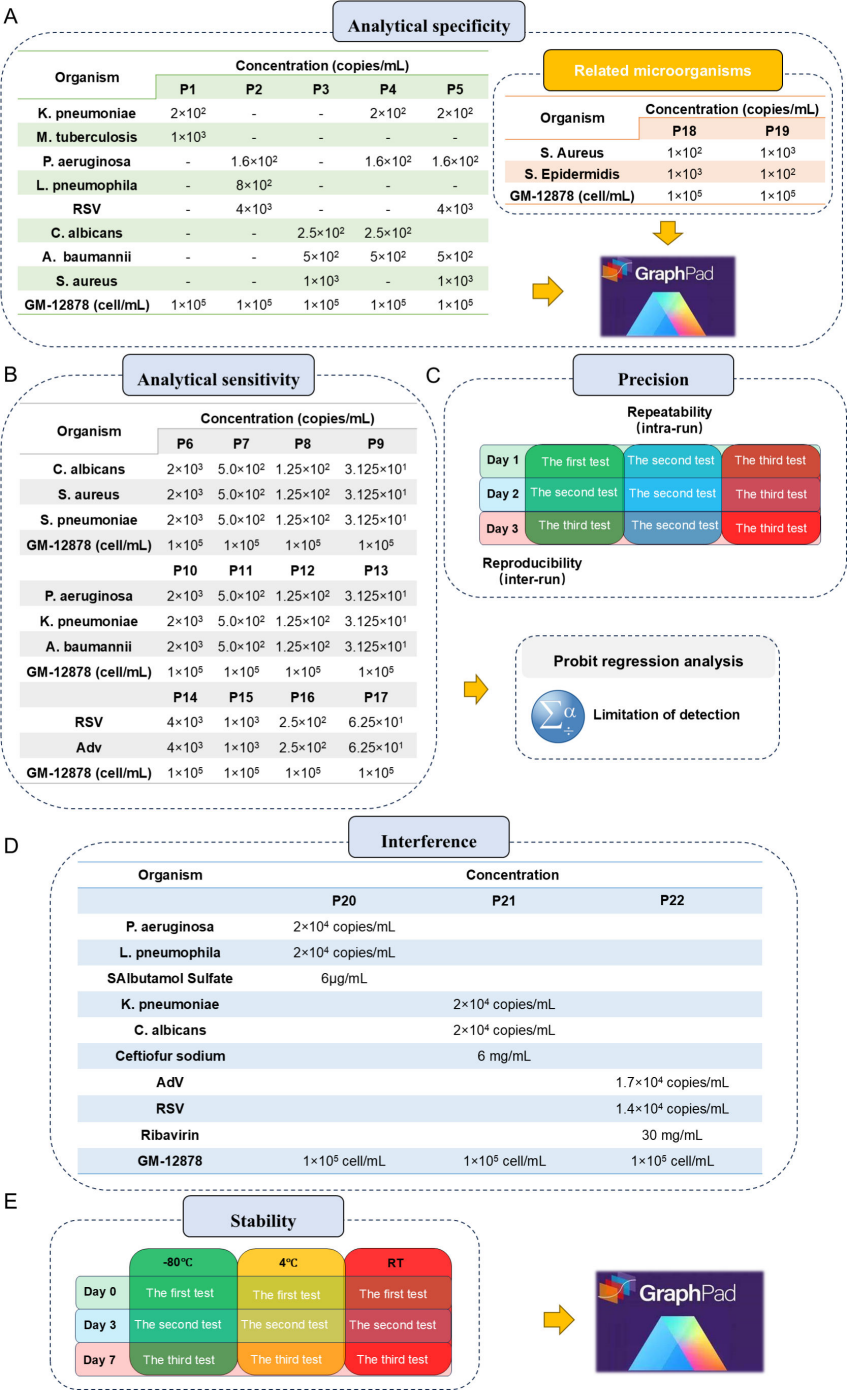
Simulated microbial sample panel for evaluating the analytical validity of tNGS. (A) Panels composition and analysis software for evaluating analytical specificity. (B) Panels composition, analysis methods, and software used to evaluate analytical sensitivity. (C) Methods for evaluating precision. (D) Panels composition for evaluating interference. (E) Methods and analysis software for evaluating stability.

A total of 22 simulated microbial sample panels were prepared to evaluate the analytical performance of tNGS (Fig. 2). P1–P5 are used to evaluate the analytical specificity of tNGS (Fig. 2A). Among them, P4 is also used to evaluate the stability of tNGS. P6–P17 are used to evaluate the analytical sensitivity of tNGS (Fig. 2B). Not only that, this study also designed P18 and P19 as the analytical specificity evaluation panels to evaluate the ability of tNGS to identify closely related microorganisms (Fig. 2A). P20–P22 are also used to evaluate the interference of drugs on tNGS (Fig. 2D). Finally, we selected *Candida albicans* and *P. aeruginosa* to form a panel for evaluating precision.

### Prepare a simulated microbial sample panel

To comprehensively evaluate the analytical detection performance of tNGS, this study uses commercialized reference materials from BeNa Culture Collection and BDS Biotechnology to prepare a simulated microbial sample panel (Table S7). The reference materials are inactivated strains or viruses, after extracted nucleic acid, detected by droplet digital PCR, and the concentration (copies per milliliter) is determined based on the number of positive droplets. Each sample panel is composed of different pathogens and diluted with phosphate-buffered saline (PBS) to the corresponding concentration. The immortal cell line (GM-12878) was quantitatively added to each panel to simulate the host background.

### Analytical specificity

There were eight common pathogens causing LRTIs (six bacteria, one virus, and one fungus) paired with each other into five sample panels (P1–P5) to evaluate the analytical specificity of tNGS (Fig. 2A). P1–P5 contain two, three, four, and five types of pathogens, respectively. Each panel was tested three times to evaluate the detection ability of tNGS against the panels that contained different numbers and concentrations of pathogens. P18 and P19 both contain S. *aureus* and *Staphylococcus epidermidis*, with concentration ratios of 1:10 and 10:1, respectively. Two panels are tested three times to evaluate the ability of tNGS to identify and detect pathogens with very similar genes when possible simultaneous infections occur (Fig. 2A).

### Analytical sensitivity

To evaluate the analytical sensitivity of as many pathogens as possible, eight representative LRTI pathogens (five bacteria, two viruses, and one fungus) were selected and combined into three different types of simulated microbial sample panels (Fig. 2B). Each type of panel was then diluted by PBS in a series with a fourfold gradient to obtain 12 panels, each of which was tested four times. Probit regression analysis is used to calculate the limit of detection (LoD) of tNGS.

### Interference

P20–P22 are used to evaluate the interference of drugs on tNGS (Fig. 2D). The three drugs that act as interfering substances are salbutamol sulfate, cefotaxime sodium, and ribavirin. Among them, salbutamol sulfate nebulized inhalation solution in P20 can be used for coughs caused by acute respiratory infections. Cefotaxime sodium in P21 is a third-generation cephalosporin that can effectively inhibit the growth and reproduction of various bacteria such as gram-positive and gram-negative bacteria, and is therefore used to treat LRTIs. Ribavirin aerosol in P22 is currently the only antiviral drug approved for the treatment of respiratory syncytial virus (RSV) infections and can also be used for the treatment of adenovirus (Adv) infections.

### Stability

To evaluate stability, P4 was divided into 27 tubes and stored at −80°C (nine tubes), 4°C (nine tubes), and room temperature (nine tubes) for 0, 3, and 7 days, respectively (Fig. 2E). At 0, 3, and 7 days, 3 P4 tubes stored at different temperatures were taken and tested using tNGS.

### Precision

To evaluate repeatability and reproducibility, the panel was divided into nine tubes and stored in a −80°C refrigerator (Fig. 2C). Three tubes were taken out daily for testing as intra-run to evaluate repeatability and tested continuously for 3 days as inter-run to evaluate reproducibility. The coefficient of variation (CV) within and between batches was calculated.

### tNGS assay

In this study, tNGS used multiplex PCR-targeted amplification technology to identify 296 clinically common pathogenic microorganisms, including bacteria, fungi, or viruses, present in the samples (Table S9). The tNGS pathogenic microorganism kit can be combined with the SansureSeq300 high-throughput sequencer to accurately detect low-concentration pathogenic microorganisms, especially their virulence and resistance genes. The chip type is flow cell high, using single-end sequencing, with a sequencing depth of approximately 1M per sample (Table S8).

The entire process of tNGS includes nucleic acid extraction and quantification, cDNA synthesis, targeted amplification, and purification of amplification products, and finally library preparation and purification. Briefly, according to the instructions of the TIANamp Yeast DNA Kit (TIANGEN, Beijing, China) (Centrifuge Column) extract pathogens nucleic acid (DNA/RNA) from each simulated microbial sample panel and clinical samples. Among them, clinical samples need to be inactivated by a high-temperature water bath. Then, the nucleic acid concentration was quantitatively detected using the Equalbit 1X dsDNA HS Assay kit (Invitrogen, Waltham, MA, USA). To detect potential RNA pathogens, a reverse transcription reaction is first performed using 5× RT Mix after nucleic acid extraction to synthesize cDNA. Multiplex PCR-targeted amplification of nucleic acids and cDNA using PO-10 Panel Mix (primer panel), 3xT Enzyme Mix (amplification mix), and IAC-PO-10 (internal reference) according to the conditions specified in the instruction. Then use the magnetic bead method to purify the amplified product. The library is prepared according to the regent instructions of the Pathogenic tNGS Library Construction Kit (296) (Sansure, Changsha, Hunan, China). Dilute the prepared library to a concentration of 1.0 ng/µL–2.0 ng/μL, with a volume of ≥10 µL. After passing the Qsep quality inspection, sequencing can be performed. The sequencing chip uses FASTASeq 300 Sequencing Flow Cell v.1.0 (Sansure, Changsha, Hunan, China). During the tNGS detection process, to eliminate false positives and contamination, three nuclease-free water samples were added as negative quality controls during nucleic acid extraction, reverse transcription, and library preparation stages, respectively. To avoid contamination, tNGS has not set up positive quality control.

### Bioinformatics analysis

Bioinformatics analysis uses a data management and analysis system (Centos 7.9). If the single-end length of the original data exceeds 50 bp, then perform low-quality filtering to retain reading Q30 >85%, ensuring high-quality data (Table S8). After single-end alignment, a self-built clinical pathogen comparison database is used to determine the read count of specific amplification targets in each sample. The reference sequences used to read the mapping are from databases from different sources, including the GenBank database, as well as from Refseq and nucleotide databases of National Center for Biotechnology Information (NCBI). The Comprehensive Antibiotic Resistance Database (CARD), Virulence Factors of Pathogenic Bacteria (VFDB) database, and the literature are used as reference databases for resistance genes and virulence genes in tNGS. Compare the detected gene sequences with the database using Burrows-Wheeler Aligner (BWA) to identify potential resistance and virulence genes. And based on their logical correlation with the pathogen, determine whether the pathogen has resistance genes and virulence genes.

### Interpretation of tNGS result

This study also classified the pathogens detected by tNGS into four categories (8). Definite: the tNGS results were consistent with the culture or thermostatic amplification gene chip method detection results conducted within 7 days after clinical collection. Probable: based on clinical, radiological, or laboratory results, the pathogen detected by tNGS may be the cause of LRTIs. Possible: the detected pathogen has pathogenic potential and is consistent with clinical presentation, but another explanation is more likely. Unlikely: the pathogen has pathogenicity but is inconsistent with clinical presentation.

### Statistical analysis

Categorical variables are expressed as percentages, while continuous variables are expressed as means and standard deviations. Calculate sensitivity, specificity, positive predictive value (PPV), and negative predictive value (NPV) at a 95% confidence interval (CI). Probit regression analysis was used to evaluate the LoD of the test results in MedCalc Statistical Software version 20.010. The consistency between tNGS and culture or composite diagnostic criteria is tested using the χ^2^ test. *P* < 0.05 is considered statistically significant; use GraphPad Prism version 9.0.0 for data analysis.

## RESULTS

This study developed a tNGS method for detecting pathogens from lower respiratory tract samples, including nucleic acid extraction, multiplex PCR-targeted amplification, library preparation, sequencing, and bioinformatics analysis. We validated the analytical and clinical performance of tNGS using simulated microbial sample panels and clinical samples with definitive microbial diagnosis. Before the start of the study, to understand the background microorganisms in the laboratory, multiple environmental samples were tested by tNGS, including the lab bench and ultra-clean bench in the laboratory reagent preparation room, the lab bench and biosafety cabinet in the sample processing room, the lab bench and biosafety cabinet in the amplification room 1, the lab bench in the amplification room 2, and the lab bench and sequencer in the sequencing room. The two main background microorganisms are *S. epidermidis* and *S. aureus*. This study used the reads per million (RPM) to perform a differential analysis of tNGS detection results. The RPM ratio of a certain microorganism is defined as the specific reads of that microorganism or the specific reads of another microorganism.

### Analytical specificity

**Fig 3:**
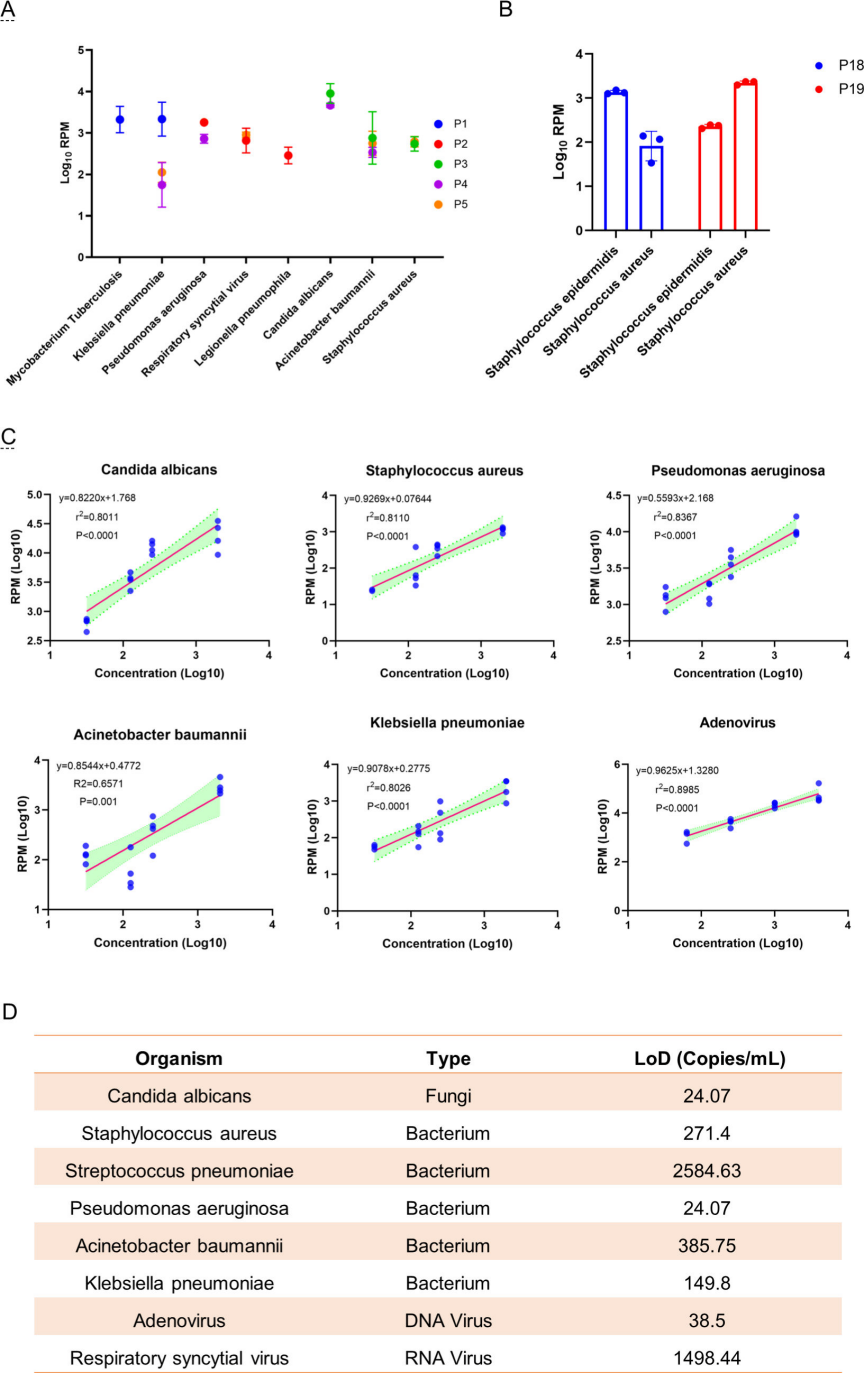
Analytical validity of tNGS. (A) Analytical specificity of tNGS. (B) The ability of tNGS to identify closely related microorganisms. (C) The relationship between the concentration of microorganisms in simulated microbial sample panel and RPM value. The *x*-axis represents log_10_ (concentration of microorganisms, with the units of copies per milliliter for DNA). The *y*-axis represents microorganisms log_10_ (RPM). The concentration of microorganisms is logarithmically linearly correlated with their RPM value. (D) The LoD of common pathogens in LRTIs estimated by probit.

Targeted amplification reagents of tNGS can amplify 296 pathogens (124 bacteria, 80 viruses, 56 fungi, 29 special pathogens, 7 genera of bacteria, 1,281 resistance genesor genotypes, and 207 virulence genes). Therefore, based on literature reports and actual clinical practice, eight common microorganisms causing LRTIs were selected and paired with each other into five different simulated microbial sample panels. The results indicate that microorganisms in P1 to P5 were successfully detected (Fig. 3A). In theory, the RPM ratios of *S. aureus* and *S. epidermidis* obtained by tNGS on P18 and P19 are approximately 1:10 and 10:1, respectively. The results show that the RPM ratios of them are 1:14 and 10:1, respectively (Fig. 3B).

### Analytical sensitivity and linearity

LoD represents the pathogen concentration when tNGS is used to repeatedly detect pathogens with a success rate of 95% (9). The result shows that the LoDs of *C. albicans*, *S. aureus*, *S. pneumoniae*, *P. aeruginosa*, *A. baumannii*, *K. pneumoniae*, Adv, and RSV were 24.07 copies/mL, 271.40 copies/mL, 2,584.63 copies/mL, 24.07 copies/mL, 385.75 copies/mL, 149.80 copies/mL, 38.50 copies/mL, and 1,498.44 copies/mL, respectively (Fig. 3D). Additionally, there was a strong linear correlation between the concentration of microorganisms and RPM value with *R*^2^ ranging from 0.66 to 0.90 (Fig. 3C). Due to the application of LoD data for linear analysis, only a portion of the data was applied, and unstable detection data near the LoD were excluded (Supplementary material).

### Precision

To evaluate the repeatability and reproducibility of tNGS, the same experimenter tested the panel three times a day for a total of 3 days. From a qualitative perspective, the results showed that the qualitative precision of tNGS was 100%. From a quantitative perspective, the repeatability (intra-run) and reproducibility (inter-run) of tNGS were evaluated; we used the RPM to calculate the CV of tNGS for detecting each pathogen (Table 1).

**TABLE 1:** Quantitative precision of tNGS assay

Organism	CV (%) of intra-run			CV (%) of inter-run
	Day 1	Day 2	Day 3	
*C. albicans*	31.94	14.61	22.92	8.76
*P. aeruginosa*	28.26	20.82	23.40	5.90

### Interference

This study evaluated the interference of tNGS by adding commonly used therapeutic drugs of LRTIs as interfering substances to simulated microbial sample panels. The results showed that interfering substances did not affect the tNGS detection of pathogens (Fig. 5B).

### Stability

**Fig 4:**
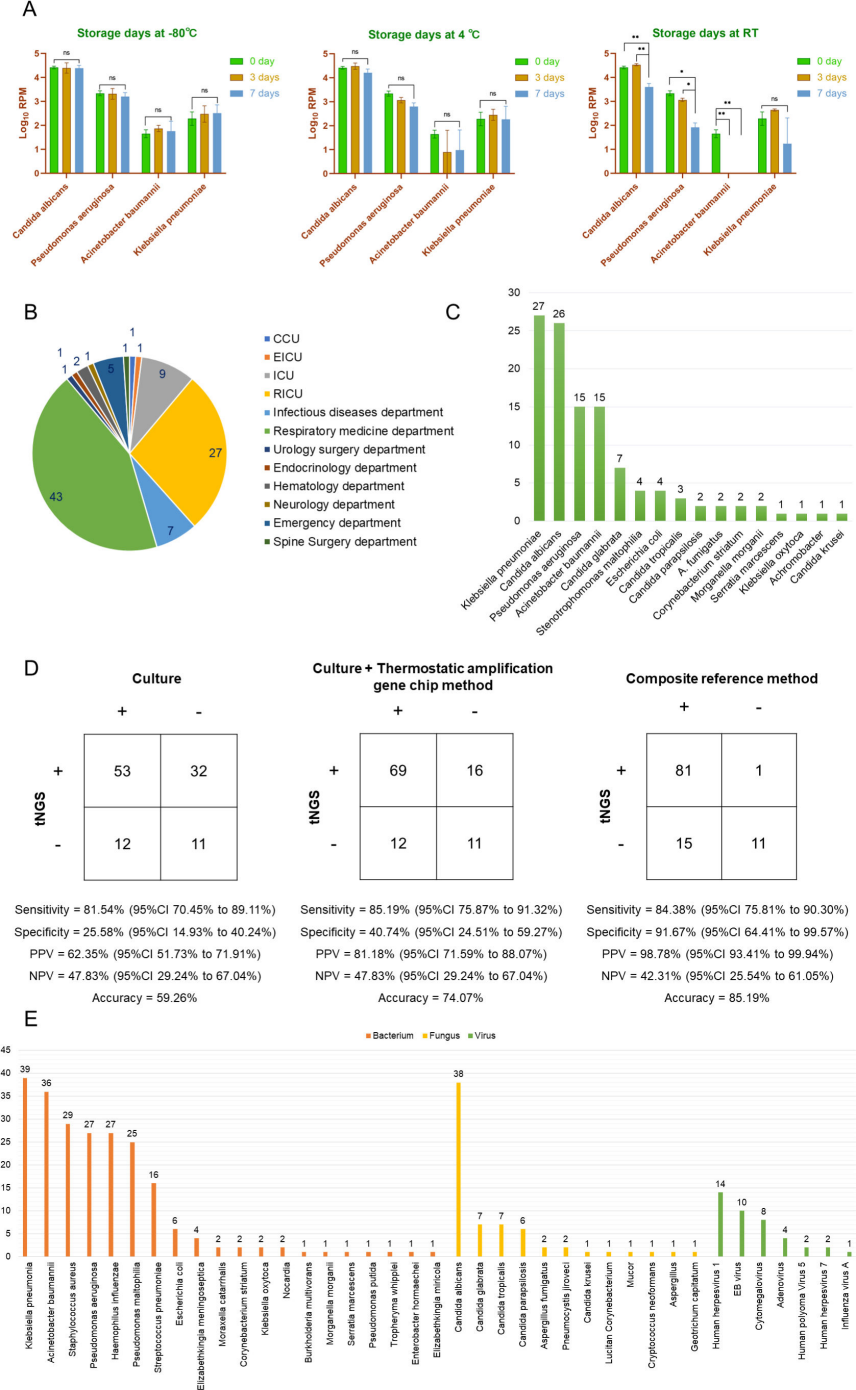
Analytical and clinical validity of tNGS. (**A**) Stability analysis of tNGS. (**B**) Departmental distribution of clinical sample sources. (**C**) The results of pathogens detected by culture. (**D**) Comparison of the performance of tNGS relative to different diagnosis criteria. (**E**) Distribution and quantity of pathogens detected by tNGS.

The results indicate that tNGS can stably detect corresponding pathogens in samples stored at −80°C and 4°C for 3 to 7 days. However, room temperature has a significant impact on tNGS detection, and the detection rate of all pathogens decreases after being stored at room temperature for 7 days (Fig. 4A).

### Patient characteristics

In this study, 108 specimens, including 101 sputum and 7 BALF, were collected from 94 diagnosed or suspected LRTI patients. The maximum and minimum ages of patients are 98 and 14 years old, respectively. The median age is 69 years old. Among them, 55 patients (58.51%) are male, and 39 patients (41.49%) are female.

### Clinical validity of tNGS

**Fig 5:**
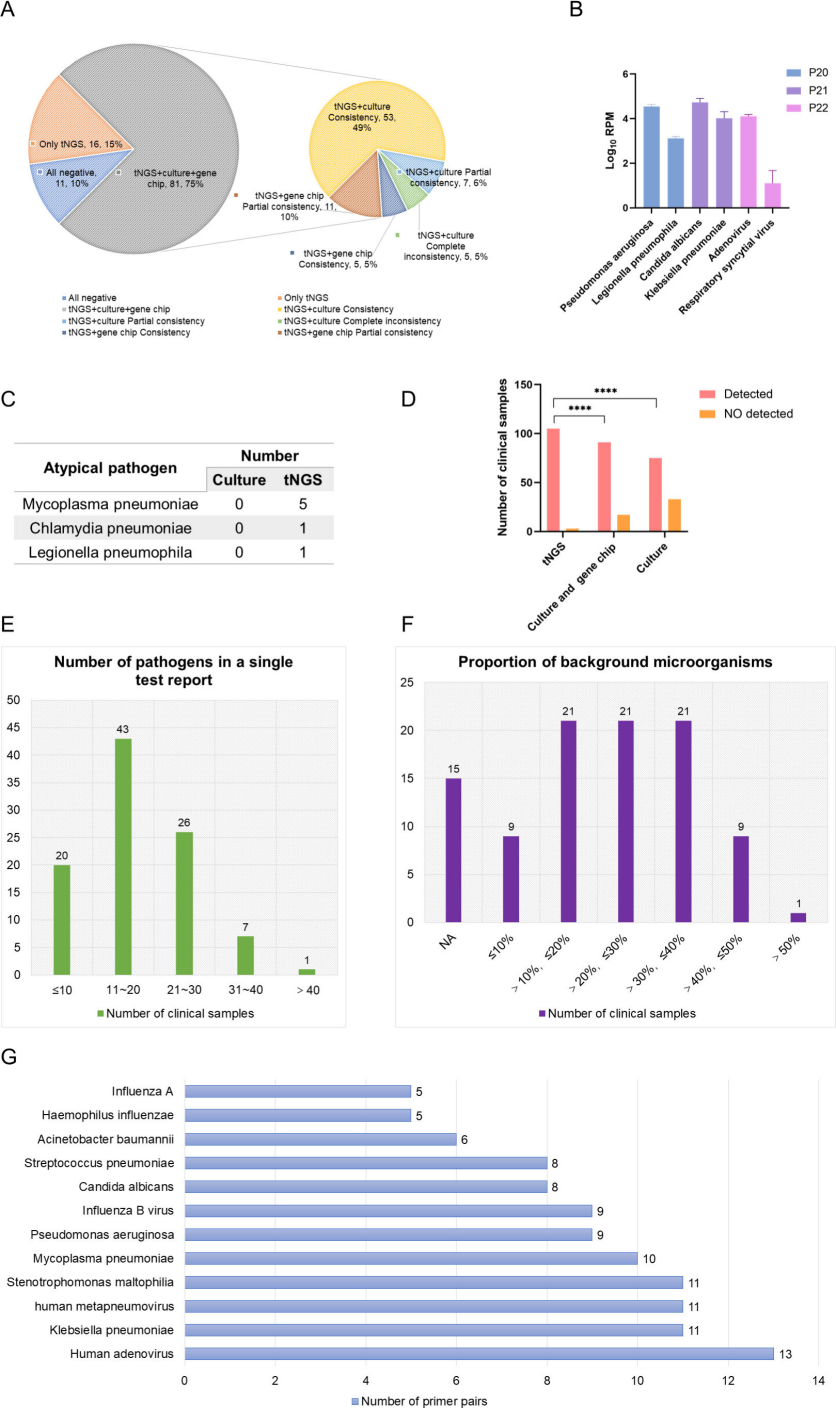
Clinical utility of tNGS for LRTI pathogen identification. (A) Consistency between tNGS results and culture results. (B) Interference of tNGS. (C) Detection of atypical pathogens by tNGS. (D) Differences in detection rates among different methods. (E) The number of pathogens in a single tNGS test report. (F) The proportion of background microorganisms in tNGS results. (G) Number of primer pairs for tNGS detection of common LRTI pathogens.

Among the 108 clinical samples, 97 were positive and 11 were negative. The conformity rates of tNGS detection results with culture, culture combined with thermostatic amplification gene chip method, and composite diagnostic criteria were 69.44% (75/108), 84.26% (91/108), and 97.22% (105/108), respectively (Fig. 5D). Using the composite diagnostic criteria as the gold standard, our in-house tNGS platform has a sensitivity of 84.38% (95% CI 75.81% to 90.30%), specificity of 91.67% (95% CI 64.41% to 99.57%), PPV of 98.78% (95% CI 93.41% to 99.94%), and NPV of 42.31% (95% CI 25.54% to 61.05%) (Fig. 4D). In this study, a total of 108 clinical samples were cultured, and 16 pathogens were identified. Among them, bacteria constituted a significant majority, representing 68.75% (11/16) of the identified pathogens. While fungi accounted for 37.50% (6/16) (Fig. 4C). The result shows that tNGS has a wider spectrum of pathogen identification than culture (Fig. 4E). tNGS detected a total of 42 pathogens, including 47.62% (20/42) bacteria, 28.57% (12/42) fungi, 16.67% (7/42) viruses, and 7.14% (3/42) atypical pathogens (Fig. 4E and 5C). The bacteria with the highest frequency of tNGS detection are *K. pneumoniae* (*n* = 39), followed by *A. baumannii* (*n* = 36), *S. aureus* (*n* = 39), *P. aeruginosa* (*n* = 27), and *H. influenzae* (*n* = 27). *C. albicans* was detected in the samples of 38 patients, which is the fungus with the highest detection rate (Fig. 5E). Among them, 11 cases of *C. albicans* were only detected by tNGS. The most common virus is human herpesvirus 1 (HHV-1), all of the seven viruses only detected by tNGS.

### Virulence and drug-resistance genes in clinical samples detected by tNGS

In this study, a total of 29 patients were tested for 25 virulence genes associated with different pathogens. Eleven virulence genes (adeF, adeG, csuD, csuE, lpsB, lpsD, lpsL, lpsM, pgaB, pgaC, and pgaD) are associated with *A. baumannii*. Ten virulence genes (clbB, clbM, iroN, ybtA, ybtE, ybtP, ybtQ, ybtS, ybtT, and ybtU) are associated with *K. pneumoniae*. Three virulence genes (exoY, fleN, and flip) are associated with *P. aeruginosa*. There is only one virulence gene (traJ) associated with *Escherichia coli*. Additionally, there are 60 patients detected 28 types of drug-resistance genes, including AAC (3´), AAC (6´), ADE, APH, ARM, CAT, cmlA, CMY, CTX-M-1, ermB, floR, KPC, mecA, mefA, msrA, NDM, OXA-1, OXA-23, OXA-51, OXA-69, QAC, SHV, TEM, tetL, tetM, tetQ, tetW, and vanA (Tables S10 and S11).

## DISCUSSION

In recent years, the NGS method has been increasingly applied in clinical practice. mNGS has shown relatively excellent performance in diagnosing LRTIs. However, we still have little understanding of the performance of tNGS, especially in terms of its analytical validity. The use of reference materials quantified by droplet digital PCR for performance validation is an important step in the NGS development process (10). This study used commercial reference materials of known concentrations to prepare simulated microbial sample panels to confirm the analytical validity of tNGS and confirmed its clinical validity using clinical samples.

In terms of analytical validity, tNGS successfully detected P1–P5 panels for evaluating analytical specificity. P1 contains two pathogens, P2 and P3 contain 3, P4 contains 4, and P5 contains 5. This indicates that tNGS can accurately detect samples containing multiple pathogens. At present, the respiratory pathogen detection reagents-based PCR method approved by the National Medical Products Administration can detect up to 13 pathogens and mainly detect viruses and mycoplasma. Similarly, the method based on a thermostatic amplification gene chip can only detect eight common bacteria. The reagents for simultaneous detection of viruses and bacteria are lacking. Compared with commonly used methods in clinical practice, tNGS has significant advantages in achieving simultaneous detection of different types of pathogens in a single detection process. Additionally, tNGS can accurately distinguish *S. aureus* and *S. epidermidis* with a concentration difference of 10 times. This also indicates that there is no interference between pathogens when tNGS detects closely related microorganisms that are coinfected.

Then, the LoD for eight common LRTI pathogens was estimated in this research. *C. albicans* and *P. aeruginosa* showed low LoD (24.07 copies/mL), while *S. pneumoniae* LoD (2,584.63 copies/mL) and RSV LoD (1,498.44 copies/mL) were higher. *C. albicans* is a thick-walled bacterium that is difficult to break. Compared to the low detection rate of fungi in mNGS, tNGS has advantages. Diao et al. estimated the LoD of mNGS for detecting *C. albicans, A. baumannii, K. pneumoniae, P. aeruginosa, S. aureus, S. pneumoniae,* and Adv, with results of 2.43, 791.12, 17.76, 127.59, 17.76, 631.29, and 1,191.18 copies/mL, respectively (11). Among them, *C. albicans*, *K. pneumoniae*, *S. aureus*, and *S. pneumoniae* were superior to tNGS in this study, but the LoDs for *A. baumannii*, *P. aeruginosa*, and Adv were inferior to those in this study. Another tNGS study suggests that the positive percent agreement of tNGS diminished when detecting bacteria and mycobacteria in the LoD range of 10^3^ to 10^4^ CFU/mL (12). This indicates that although the target sequence has been enriched, it cannot be reliably detected by tNGS when the pathogen concentration is close to or below 10^3^ CFU/mL. Many factors affect the analytical sensitivity of NGS, which may involve all steps from sample collection to bioinformatics analysis (13). For tNGS, it is also necessary to consider that the possible interactions between primers in multiplex PCR are important factors affecting LoD (14). However, compared to the LoD of 500 copies/mL of PCR-based assay kits used in clinical practice, tNGS still has advantages. It should be emphasized here that P14–P17 include Adv and RSV, the former being DNA virus and the latter being RNA virus. This indirectly confirms that tNGS can detect both DNA and RNA viruses simultaneously, and compared to mNGS, which requires separate processing of DNA and RNA, the operation process of tNGS is simpler.

The qualitative precision of this study reached 100%, which is consistent with the CV detected by other mNGS studies (9). Blauwkamp et al. reported that the CV of mNGS tests for microbial cell-free DNA (cfDNA) ranged from 16.7 to 18.9% within a run and from 17.9 to 22.2% within the laboratory (15). Similarly, Chen et al.’s research shows that for cell-free DNA, the CV of mNGS for all microorganisms ranged from 11.2% to 18.4% in the intra-run and from 16.5% to 25.0% in the inter-run (16). For the cellular DNA method, the CV for all microorganisms ranged from 12.8% to 18.2% in the intra-run and from 18.5% to 23.4% in the inter-run. Diao et al. found that the CV of mNGS for all microorganisms ranged from 17.39% to 38.16% within a run and from 16.36% to 38.17% within the laboratory (11). Our results are similar to this. Compared to cfDNA, the nucleic acid extraction process has been proven to be one of the sources of variation (17). Moreover, the selection of PCR amplification regions, primer design, and the number of amplification cycles may all be the reasons for the increase in CV differences among different methods. Consequently, tNGS is more suitable for qualitative detection.

In terms of clinical validity, the results of this study indicate that tNGS can effectively detect common pathogens that cause LRTIs, such as *S. pneumoniae*, *H. influenzae*, *Moraxella catarrhalis*, *S. aureus*, and other G^-^ bacteria (Fig. 4; Table S12). The detection rates of *S. pneumoniae* (0.93% [1/108] vs 14.81% [16/108], *P* = 0.0002) and *H. influenzae* (12.96% [14/108] vs 25% [27/108], *P* = 0.0241) were most significant compared to cultivation. Moreover, tNGS has significant advantages in fungal detection; it can simultaneously detect multiple fungal infections in patients, such as *C. albicans* combined with *Candida parapsilosis* infection.

Among the pathogens that cause LRTIs, viruses are crucial and difficult to identify. This study detected 14 cases of HHV-1, 10 cases of Epstein-Barr (EB) virus, 8 cases of cytomegalovirus, 4 cases of Adv, 2 cases of human polyomavirus 5, 2 cases of HHV-7, and 1 case of influenza A virus (Fig. 4E). Similar to other studies, this study also confirms that patients in the intensive care unit (ICU) are often infected with HHV, cytomegalovirus, or EB virus (18–20). It is worth noting that no corresponding pathogen was detected in the samples of one patient with coronavirus disease 2019 and one patient with influenza B virus. This may be because the RNA virus is very easy to degrade, and freezing and thawing may occur during sample transportation and storage, which reduces the detection rate of the RNA virus. In 2024, WHO defines influenza as non-severe and severe influenza (18). Some non-severe influenza patients with significant risk factors may develop into severe influenza, which can cause serious complications and even death. Therefore, timely identification and intervention of such patients can avoid serious consequences. However, false-negative results cannot provide timely information for clinical management decisions (18). To avoid false-negative results, several issues need to be noted during sample collection, transportation, and storage. Samples should be collected within 3–10 days after the first appearance of symptoms (19). Antiviral drugs can keep the viral load low, which may lead to false-negative results (20). Thus, it is necessary to collect samples before taking antiviral drugs. The sample collection methodology directly affects the detection of viral load, so the collection process should be carried out by trained professionals (19). Additionally, samples should be transported in a three-layer packaging consisting of leakproof containers in a low-temperature environment (21). Bad storage samples also can lead to RNA degradation, resulting in false-negative results (22). After collection, store at 2–8°C for no more than 72 h. Samples must be stored at −70°C or below during testing or transportation delays (19). Despite achieving these, molecular diagnostic experts need to communicate with clinical physicians and combine tNGS test results with actual clinical conditions. Once there is a discrepancy between the two, other tests such as nucleic acid amplification testing, antigen-antibody testing, microbial culture, and C-reactive protein can be improved for a comprehensive evaluation to alleviate the impact of false-negative results on patients.

Compared to other microbial detection methods, tNGS has the following three advantages. Firstly, the advantages of tNGS in detecting atypical pathogens have expanded the detection range of the pathogen spectrum (Fig. 5C). Pneumonia caused by *Mycoplasma pneumoniae* cannot be diagnosed solely based on clinical presentation and symptoms (23). However, due to the lack of cell walls and slow growth, it is challenging to detect through culture. The incidence rate of *Pneumocystis jiroveci* increases in patients with immunocompromised, it has the characteristics of rapid progression, critical ill, and poor prognosis (24). Timely diagnosis and target treatment are the keys to reducing its mortality rate. However, early diagnosis is difficult and easily leads to delays in treatment. Similarly, *Cryptococcus neoformans* also has the problem of long cultivation time and low positivity rate (25). This study confirms that tNGS, as a new tool, effectively compensates for the shortcomings of traditional methods such as culture and enhances the ability of the laboratory to detect and identify atypical pathogens. Furthermore, it is always difficult to treat hospital-acquired pneumonia (HAP) and ventilator-associated pneumonia (VAP), and the problem of high mortality and morbidity always exists (26). Compared to others, patients in the ICU face more particular risks and are more prone to HAP (27). Among them, over 90% of cases occur in patients who are intubated and mechanically ventilated (28). In this study, samples from ICU, respiratory intensive care unit (RICU), coronary care unit (CCU), and emergency intensive care unit (EICU) accounted for 34.26% (37/108) of the total samples. Community and hospital-acquired LRTI is believed to be caused by the expansion of the range of single and multiple microbial infections (29). tNGS detected only one pathogen in 16 samples, including 2 RICU samples and 1 ICU sample, while multiple pathogens were detected in the remaining 81 samples. The latest guidelines in Europe suggest that patients with such infections must receive appropriate empirical antibiotic treatment as soon as possible (30). However, inappropriate empirical antibiotic treatment may lead to higher mortality rates in ICU patients (31). This study also confirms that tNGS has significant advantages in detecting superinfection and coinfection, especially for ICU patients. Finally, similar to mNGS, the advantage of tNGS is also helpful in diagnosing patients with negative culture results but suspected infections (29). In this study, 14 patients were found to have no pathogens detected through culture and thermostatic amplification gene chip. According to tNGS detection, 5 of them were diagnosed with infection with *H. influenzae*, 5 *S. pneumoniae*, 2 *S. aureus*, 1 *C. albicans*, and 1 *A. baumannii*.

However, it should be emphasized that similar to mNGS, tNGS currently also lacks standards for interpreting results and cannot be used to confirm diagnosis. They can only serve as auxiliary methods for screening causative pathogens. Especially for sputum samples, the result of tNGS presents a large number of conditioned pathogens and normal flora, which makes it extremely challenging to determine the pathogen of LRTIs. The pathogens detected by tNGS can only represent the presence of these microorganisms in the specimen, but it cannot be determined whether they are pathogenic bacteria. Once misidentified, it may lead to the misuse of antibiotics. Thus, it is necessary to comprehensively evaluate the type of pathogenic microorganisms, the immune status of the patient, clinical symptoms, and other factors when interpreting the results. Gaston et al. found that the reported number of pathogens in a single mNGS test ranged from 4 to 24, with tNGS concentrated in 1 to 2 (12). However, in this study, the number of pathogens reported ranged from 4 to 45 in a single tNGS test (Fig. 5E). Additionally, after tNGS automatic bioinformatics analysis, the proportion of background microorganisms results of 97 samples ranged from 6.25% to 54.17%. (Fig. 5F).

In addition to a limited number of drug-resistance genes, more research is needed to elucidate the relationship between resistance genes and phenotypes if tNGS is to be used as a basis for drug resistance in clinical applications. From another perspective, primers designed by tNGS for drug-resistance genes can not only be used to indicate the drug-resistance status of pathogens but also to further confirm the existence of the pathogen in the sample, providing a basis for pathogen identification.

In terms of clinical utility, the sample TAT of tNGS is 24 h, which is significantly shorter than the 3–5 days of conventional culture methods. Thus, tNGS can quickly and accurately identify pathogens without losing sensitivity and has high application value for early diagnosis and treatment of LRTIs. At present, the sequencing depth of mNGS mainly depends on expected results and budget, without hard and clear rules (32). For example, for the diagnosis of LRTIs, sequencing depths range from 2M to 25M. When the depth is below 5M, false-negative results may occur due to insufficient depth. As the sequencing depth increases, the analytical sensitivity will also be higher, but the cost will also increase. However, the sequencing depth of tNGS is approximately 1M, ensuring accuracy while reducing costs. tNGS can help patients with negative culture and other microbiological tests receive early diagnosis and can serve as a preliminary screening method for identifying LRTI pathogens. This study also found that tNGS can help detect different pathogens in the same sample of ICU or RICU patients with long hospital stays and immunocompromised, further shortening the average hospital stay, reducing the economic burden on patients, and reducing the waste of medical resources.

### Limitation and perspective

However, although we tried our best to design a comprehensive study, there are still limitations. In the performance validation of the analytical specificity of tNGS, evaluating whether all pathogens can be accurately detected not only requires a huge amount of work but also may face difficulties such as a lack of reference materials or corresponding clinical samples for pathogens. Therefore, this study only confirmed the detection of common pathogens causing LRTI by tNGS.

Similar to mNGS, tNGS also requires the establishment of quality control standards and standardized operating procedures. Unlike mNGS, tNGS is a technology established based on multiplex PCR. tNGS utilizes a large number of primers designed for a pathogen in its multiplex PCR reaction (Fig. 5G). Therefore, in order to ensure detection specificity, it is necessary to optimize the primers for microorganisms that are prone to mutations in a timely manner, to ensure accurate and reliable detection results and reduce non-specific amplification. For example, primers and probes designed for RNA virus genomes may be affected by mutation sites. Thus, it is recommended to select multiple regions within the conserved regions of the viral genome as target areas for primer design (33). When selecting the target area for primer design, it is necessary to align the genome sequence and remove regions highly homologous to other pathogens to ensure primer specificity (34). After the primer design is completed, primer specificity and cross-reactivity can be validated through bioinformatics and experimental methods. Additionally, during primer optimization, it is essential to consider the mutual influence between primers to avoid the sensitivity of detection being affected by primer dimerization or amplification bias. Primers designed for different target regions may have significant differences in sensitivity, which are further amplified during the amplification process, ultimately leading to PCR bias (35). Therefore, in primer design, in addition to the length of the primer, CG content, melting temperature, avoidance of mutation sites, avoidance of secondary structures, and primer dimers, the amplification efficiency and sensitivity of the primer should also be verified, and primers that are similar to both should be selected as much as possible (14). In the future, molecular diagnostic experts can enhance communication and cooperation with clinical physicians, customize tNGS detection panels for different diseases, and ensure that they exhibit excellent analytical and clinical performance.

### Conclusions

In summary, this study utilized reference materials to prepare simulated microbial sample panels and developed a comprehensive validation protocol to assess the analytical validity of tNGS in diagnosing LRTIs, providing a ready-made example for clinical use. By examining the clinical validity of tNGS using actual clinical samples, it was found that tNGS can reliably detect common pathogens in LRTIs, including identifying co-infections and atypical pathogens. From the perspective of clinical utility, tNGS also significantly reduces the TAT. Following thorough validation, the tNGS workflow has performed well and is expected to be utilized in clinical practice to aid in the diagnosis of LRTIs and patient management.

## Data Availability

The raw data has been stored in the NCBI Sequence Read Archive (SRA) database (http://www.ncbi.nlm.nih.gov/sra), accession number PRJNA1263143.

## SUPPLEMENTAL MATERIAL

Supplemental Tables spectrum.01751-24-s0001.docx Tables S1-S12.

ASM does not own the copyrights to Supplemental Material that may be linked to, or accessed through, an article. The authors have granted ASM a non-exclusive, world-wide license to publish the Supplemental Material files. Please contact the corresponding author directly for reuse.
