# Supplementary material for: Laboratory validation of targeted next-generation sequencing assay for pathogen detection in lower respiratory infection
Source: Microbiol Spectr. 2025 May 21;13(7):e01751-24. doi: 10.1128/spectrum.01751-24 (PMC12210981; doi:10.1128/spectrum.01751-24)
Supplement: Supplemental Tables — Tables S1-S12. [file spectrum.01751-24-s0001.docx]

**Supplementary material for “****Laboratory validation of** **targeted next-generation sequencing assay for pathogen detection in** **lower respiratory infection”**

**Table S1. Analytical specificity results of tNGS**

| **Sample panel** | **Organism** | **Reads count** | **Correctly matched reads** | **Situation of each primer** |
| --- | --- | --- | --- | --- |
| P1-1 | M. tuberculosis complex | 13901 | 1568691 | 5/5 |
|  | M. tuberculosis | 2149 | 1568691 | 10/15 |
|  | K. pneumoniae | 1167 | 1568691 | 5/11 |
| P1-2 | K. pneumoniae | 1325 | 431604 | 5/11 |
|  | M. tuberculosis complex | 3617 | 431604 | 5/5 |
|  | M. tuberculosis | 592 | 431604 | 9/15 |
| P1-3 | M. tuberculosis complex | 6637 | 147454 | 5/5 |
|  | M. tuberculosis | 728 | 147454 | 9/15 |
|  | K. pneumoniae | 639 | 147454 | 5/11 |
| P2-1 | P. aeruginosa | 3254 | 1525191 | 8/9 |
|  | RSV B | 2132 | 1525191 | 3/5 |
|  | L. pneumophila | 727 | 1525191 | 4/5 |
| P2-2 | P. aeruginosa | 5742 | 3810881 | 8/9 |
|  | RSV B | 1457 | 3810881 | 3/5 |
|  | L. pneumophila | 742 | 3810881 | 4/5 |
| P2-3 | P. aeruginosa | 3253 | 1830131 | 8/9 |
|  | RSV B | 971 | 1830131 | 3/5 |
|  | L. pneumophila | 458 | 1830131 | 4/5 |
| P3-1 | C. albicans | 17480 | 1432860 | 3/8 |
|  | A. baumannii | 2603 | 1432860 | 4/6 |
|  | S. aureus | 1169 | 1432860 | 2/8 |
| P3-2 | C. albicans | 21869 | 1761659 | 3/8 |
|  | A. baumannii | 2982 | 1761659 | 4/6 |
|  | S. aureus | 974 | 1761659 | 2/8 |
| P3-3 | C. albicans | 8065 | 1687545 | 3/8 |
|  | S. aureus | 617 | 1687545 | 2/8 |
|  | A. baumannii | 238 | 1687545 | 4/6 |
| P4-1 | C. albicans | 17228 | 3502035 | 3/8 |
|  | P. aeruginosa | 2780 | 3502035 | 8/9 |
|  | K. pneumoniae | 610 | 3502035 | 5/11 |
|  | A. baumannii | 1259 | 3502035 | 4/6 |
| P4-2 | C. albicans | 14049 | 2818410 | 3/8 |
|  | P. aeruginosa | 2433 | 2818410 | 8/9 |
|  | A. baumannii | 1241 | 2818410 | 4/6 |
|  | K. pneumoniae | 191 | 2818410 | 5/11 |
| P4-3 | C. albicans | 14693 | 3601323 | 3/8 |
|  | P. aeruginosa | 1983 | 3601323 | 8/9 |
|  | A. baumannii | 899 | 3601323 | 4/6 |
|  | K. pneumoniae | 53 | 3601323 | 4/11 |
| P5-1 | P. aeruginosa | 2664 | 1474156 | 8/9 |
|  | RSV B | 1506 | 1474156 | 3/5 |
|  | A. baumannii | 1478 | 1474156 | 4/6 |
|  | S. aureus | 1255 | 1474156 | 2/8 |
|  | K. pneumoniae | 315 | 1474156 | 5/11 |
| P5-2 | P. aeruginosa | 3633 | 2041901 | 8/9 |
|  | RSV B | 1909 | 2041901 | 3/5 |
|  | A. baumannii | 1360 | 2041901 | 4/6 |
|  | S. aureus | 1016 | 2041901 | 2/8 |
|  | K. pneumoniae | 171 | 2041901 | 5/11 |
| P5-3 | P. aeruginosa | 3247 | 1725897 | 8/9 |
|  | RSV B | 1306 | 1725897 | 3/5 |
|  | S. aureus | 958 | 1725897 | 2/8 |
|  | A. baumannii | 469 | 1725897 | 4/6 |
|  | K. pneumoniae | 137 | 1725897 | 5/11 |

**Table S2. Analysis specificity results of tNGS (Closely related microorganisms)**

| **Sample panel** | **Organism** | **Reads count** | **Correctly matched reads** | **Situation of each primer** |
| --- | --- | --- | --- | --- |
| P18-1 | S. aureus | 23 | 685912 | 2/8 |
|  | S.epidermidis | 866 | 685912 | 3/3 |
| P18-2 | S. aureus | 103 | 752666 | 2/8 |
|  | S.epidermidis | 1010 | 752666 | 3/3 |
| P18-3 | S. aureus | 90 | 761000 | 3/8 |
|  | S.epidermidis | 1142 | 761000 | 3/3 |
| P19-1 | S. aureus | 1551 | 657177 | 2/8 |
|  | S.epidermidis | 157 | 657177 | 3/3 |
| P19-2 | S. aureus | 1099 | 464098 | 2/8 |
|  | S.epidermidis | 113 | 464098 | 3/3 |
| P19-3 | S. aureus | 1316 | 623930 | 2/8 |
|  | S.epidermidis | 126 | 623930 | 3/3 |

**Table S3. Interference results of tNGS**

| **Sample panel** | **Organism** | **Reads count** | **Correctly matched reads** | **Situation of each primer** |
| --- | --- | --- | --- | --- |
| P20-1 | P. aeruginosa | 37324 | 857022 | 8/9 |
|  | L. pneumophila | 1394 | 857022 | 4/5 |
| P20-2 | P. aeruginosa | 26639 | 899036 | 8/9 |
|  | L. pneumophila | 975 | 899036 | 4/5 |
| P20-3 | P. aeruginosa | 28863 | 870153 | 8/9 |
|  | L. pneumophila | 1095 | 870153 | 4/5 |
| P21-1 | C. albicans | 26754 | 314626 | 3/8 |
|  | K. pneumoniae | 7018 | 314626 | 5/11 |
| P21-2 | C. albicans | 38706 | 1024513 | 3/8 |
|  | K. pneumoniae | 6364 | 1024513 | 5/11 |
| P21-3 | C. albicans | 32639 | 699818 | 3/8 |
|  | K. pneumoniae | 5707 | 699818 | 5/11 |
| P22-1 | Adv | 8822 | 555811 | 6/13 |
|  | RSV B | 28 | 555811 | 2/5 |
| P22-2 | Adv | 9021 | 825649 | 6/13 |
|  | RSV B | 9 | 825649 | 2/5 |
| P22-3 | Adv | 6513 | 546576 | 6/13 |
|  | RSV B | 2 | 546576 | 1/5 |

**Table S4. Analysis sensitivity results of tNGS**

| **Concentration**  **(copies/mL)** | **Number of positive duplicates/total number of test duplicates for each dilution (positive rate, %)** | | | | | | | |
| --- | --- | --- | --- | --- | --- | --- | --- | --- |
|  | **C. albicans** | **S. aureus** | **S. pneumoniae** | **P. aeruginosa** | **A. baumannii** | **K. pneumoniae** | **Adv** | **RSV B** |
| 2000 | 4/4 (100%) | 4/4 (100%) | 3/4 (75%) | 4/4 (100%) | 4/4 (100%) | 4/4 (100%) | 4/4 (100%) | 4/4 (100%) |
| 500 | 4/4 (100%) | 4/4 (100%) | 2/4 (50%) | 4/4 (100%) | 4/4 (100%) | 4/4 (100%) | 4/4 (100%) | 0/4 (0%) |
| 125 | 4/4 (100%) | 1/4 (25%) | 0/4 (0%) | 4/4 (100%) | 1/4 (25%) | 3/4 (75%) | 4/4 (100%) | 0/4 (0%) |
| 31.25 | 4/4 (100%) | 0/4 (0%) | 0/4 (0%) | 4/4 (100%) | 1/4 (25%) | 0/4 (0%) | 3/4 (75%) | 0/4 (0%) |
| 0 | 0/4 (0%) | 0/4 (0%) | 0/4 (0%) | 0/4 (0%) | 0/4 (0%) | 0/4 (0%) | 0/4 (0%) | 0/4 (0%) |

**Table S5. Detection rate of pathogens by tNGS under different storage conditions and storage times**

| Storage time | Storage temperature | Detection rate of each bacterium detected three times | | | |
| --- | --- | --- | --- | --- | --- |
|  |  | C. albicans | P. aeruginosa | K. pneumoniae | A. baumannii |
| Day 0 | - | 3/3 (100%) | 3/3 (100%) | 3/3 (100%) | 3/3 (100%) |
| Day 3 | RT | 3/3 (100%) | 3/3 (100%) | 3/3 (100%) | 0/3 (0%) |
|  | 4℃ | 3/3 (100%) | 3/3 (100%) | 3/3 (100%) | 1/3 (33%) |
|  | -80℃ | 3/3 (100%) | 3/3 (100%) | 3/3 (100%) | 0/3 (0%) |
| Day 7 | RT | 3/3 (100%) | 1/3 (33%) | 1/3 (33%) | 0/3 (0%) |
|  | 4℃ | 3/3 (100%) | 3/3 (100%) | 2/3 (67%) | 1/3 (33%) |
|  | -80℃ | 3/3 (100%) | 3/3 (100%) | 3/3 (100%) | 0/3 (0%) |

**Table S6. Liner regression and correlation result of tNGS**

| **Organism** | **Concentration (Log10)** | **Reads count** | **Correctly matched reads** | **RPM**  **(Log10)** | **Organism** | **Concentration (Log10)** | **Reads count** | **Correctly matched reads** | **RPM**  **(Log10)** |
| --- | --- | --- | --- | --- | --- | --- | --- | --- | --- |
| C. albicans | 3.3 | 17548 | 44114.95 | 4.64 | S. aureus | 3.3 | 358 | 900.00 | 2.95 |
| C. albicans | 3.3 | 20273 | 30402.87 | 4.48 | S. aureus | 3.3 | 814 | 1220.73 | 3.09 |
| C. albicans | 3.3 | 28547 | 35727.25 | 4.55 | S. aureus | 3.3 | 943 | 1180.19 | 3.07 |
| C. albicans | 3.3 | 18806 | 27047.08 | 4.43 | S. aureus | 3.3 | 907 | 1304.46 | 3.12 |
| C. albicans | 2.4 | 9022 | 16404.20 | 4.21 | S. aureus | 2.4 | 190 | 345.47 | 2.54 |
| C. albicans | 2.4 | 5558 | 9377.25 | 3.97 | S. aureus | 2.4 | 126 | 212.58 | 2.33 |
| C. albicans | 2.4 | 6373 | 11321.55 | 4.05 | S. aureus | 2.4 | 235 | 417.47 | 2.62 |
| C. albicans | 2.4 | 8333 | 14136.07 | 4.15 | S. aureus | 2.4 | 262 | 444.46 | 2.65 |
| C. albicans | 2.1 | 2354 | 3694.38 | 3.57 | S. aureus | 2.1 | 21 | 32.96 | 1.52 |
| C. albicans | 2.1 | 1629 | 2180.79 | 3.34 | S. aureus | 2.1 | 47 | 62.92 | 1.8 |
| C. albicans | 2.1 | 1788 | 3465.97 | 3.54 | S. aureus | 2.1 | 196 | 379.94 | 2.58 |
| C. albicans | 2.1 | 2877 | 4689.40 | 3.67 | S. aureus | 2.1 | 32 | 52.16 | 1.72 |
| C. albicans | 1.5 | 258 | 443.24 | 2.65 | S. aureus | 1.5 | 15 | 25.77 | 1.41 |
| C. albicans | 1.5 | 431 | 743.05 | 2.87 | S. aureus | 1.5 | 12 | 23.43 | 1.37 |
| C. albicans | 1.5 | 349 | 681.48 | 2.83 | S. aureus | 1.5 | - | - | - |
| C. albicans | 1.5 | 349 | 698.18 | 2.84 | S. aureus | 1.5 | - | - | - |
| P. aeruginosa | 3.3 | 9680 | 10008.18 | 4.00 | K. pneumoniae | 3.3 | 841 | 869.51 | 2.94 |
| P. aeruginosa | 3.3 | 8453 | 9064.02 | 3.96 | K. pneumoniae | 3.3 | 1649 | 1768.20 | 3.25 |
| P. aeruginosa | 3.3 | 14842 | 16393.99 | 4.21 | K. pneumoniae | 3.3 | 3129 | 3456.19 | 3.54 |
| P. aeruginosa | 3.3 | 8139 | 9613.09 | 3.98 | K. pneumoniae | 3.3 | 2943 | 3476.02 | 3.54 |
| P. aeruginosa | 2.4 | 1818 | 2398.28 | 3.38 | K. pneumoniae | 2.4 | 101 | 133.24 | 2.12 |
| P. aeruginosa | 2.4 | 2241 | 3553.76 | 3.55 | K. pneumoniae | 2.4 | 56 | 88.80 | 1.95 |
| P. aeruginosa | 2.4 | 3511 | 5607.81 | 3.75 | K. pneumoniae | 2.4 | 298 | 475.97 | 2.68 |
| P. aeruginosa | 2.4 | 4120 | 4418.65 | 3.65 | K. pneumoniae | 2.4 | 917 | 983.47 | 2.99 |
| P. aeruginosa | 2.1 | 1105 | 1900.46 | 3.28 | K. pneumoniae | 2.1 | 86 | 147.91 | 2.17 |
| P. aeruginosa | 2.1 | 1310 | 1928.80 | 3.29 | K. pneumoniae | 2.1 | 142 | 209.08 | 2.32 |
| P. aeruginosa | 2.1 | 577 | 1014.66 | 3.01 | K. pneumoniae | 2.1 | 71 | 124.85 | 2.10 |
| P. aeruginosa | 2.1 | 819 | 1194.16 | 3.08 | K. pneumoniae | 2.1 | 38 | 55.41 | 1.74 |
| P. aeruginosa | 1.5 | 241 | 792.68 | 2.90 | K. pneumoniae | 1.5 | 15 | 63.72 | 1.80 |
| P. aeruginosa | 1.5 | 314 | 1333.91 | 3.13 | K. pneumoniae | 1.5 | 16 | 56.65 | 1.75 |
| P. aeruginosa | 1.5 | 350 | 1239.23 | 3.09 | K. pneumoniae | 1.5 | 14 | 48.34 | 1.68 |
| P. aeruginosa | 1.5 | 506 | 1747.29 | 3.24 | K. pneumoniae | 1.5 | - | - | - |
| A. baumannii | 3.3 | 2369 | 2449.32 | 3.39 | Adenovirus | 3.6 | 27113 | 166178.38 | 5.22 |
| A. baumannii | 3.3 | 2639 | 2829.76 | 3.45 | Adenovirus | 3.6 | 24055 | 43056.54 | 4.63 |
| A. baumannii | 3.3 | 4156 | 4590.58 | 3.66 | Adenovirus | 3.6 | 15615 | 34383.14 | 4.54 |
| A. baumannii | 3.3 | 1831 | 2162.62 | 3.33 | Adenovirus | 3.6 | 25388 | 32526.82 | 4.51 |
| A. baumannii | 2.4 | 314 | 414.23 | 2.62 | Adenovirus | 3 | 8557 | 26525.68 | 4.42 |
| A. baumannii | 2.4 | 304 | 482.08 | 2.68 | Adenovirus | 3 | 6125 | 20491.12 | 4.31 |
| A. baumannii | 2.4 | 463 | 739.51 | 2.87 | Adenovirus | 3 | 7976 | 25668.83 | 4.41 |
| A. baumannii | 2.4 | 113 | 121.19 | 2.08 | Adenovirus | 3 | 7472 | 15409.59 | 4.19 |
| A. baumannii | 2.1 | 104 | 178.87 | 2.25 | Adenovirus | 2.4 | 726 | 2318.82 | 3.37 |
| A. baumannii | 2.1 | 23 | 33.86 | 1.53 | Adenovirus | 2.4 | 1491 | 5571.06 | 3.75 |
| A. baumannii | 2.1 | 16 | 28.14 | 1.45 | Adenovirus | 2.4 | 1858 | 4388.08 | 3.64 |
| A. baumannii | 2.1 | 36 | 52.49 | 1.72 | Adenovirus | 2.4 | 1877 | 4569.04 | 3.66 |
| A. baumannii | 1.5 | 39 | 128.28 | 2.11 | Adenovirus | 1.8 | 181 | 546.82 | 2.74 |
| A. baumannii | 1.5 | 29 | 123.20 | 2.09 | Adenovirus | 1.8 | 600 | 1427.53 | 3.15 |
| A. baumannii | 1.5 | 23 | 81.44 | 1.91 | Adenovirus | 1.8 | 454 | 1646.24 | 3.22 |
| A. baumannii | 1.5 | 55 | 189.92 | 2.28 | Adenovirus | 1.8 | - | - | - |

**Table S7. Basic information of reference materials**

| **Pathogens** | **Item number** | **Manufacturer** | **Concentration** |
| --- | --- | --- | --- |
| K. pneumoniae | BNCC 363157 | BeNa Culture Collection | 2.73×10^6^ copies/mL |
| M. tuberculosis | BNCC 363310 | BeNa Culture Collection | 4.30×10^8^ CFU/mL |
| P. aeruginosa | BNCC 363465 | BeNa Culture Collection | 5.10×10^6^ copies/mL |
| L. pneumophila | BNCC 364348 | BeNa Culture Collection | 5.10×10^6^ copies/mL |
| RSV | BDS-IQC-230 | BDS | 1.4×10^4^ copies/mL |
| C. albicans | BNCC 364342 | BeNa Culture Collection | 3.83×10^6^ copies/mL |
| A. baumannii | BNCC 363270 | BeNa Culture Collection | 3.60 ×10^6^ copies/mL |
| S. aureus | BNCC 364341 | BeNa Culture Collection | 6.40×10^6^ copies/mL |
| S. pneumoniae | BNCC 338425 | BeNa Culture Collection | 4.70×10^6^ copies/mL |
| Adv | BDS-IQC-233 | BDS | 1.7×10^4^ copies/mL |

**Table S8. The information of chip specification of FCH**

| **Chip specifications** | **Pipeline** | **Reads** | **sequencing strategy** | | **Data product (Gb)** | | **Q30** | | **Sequencing time index (0)** | | **Sequencing time**  **index (8+8)** | |
| --- | --- | --- | --- | --- | --- | --- | --- | --- | --- | --- | --- | --- |
| **Flow Cell High**  **(FCH)** | **4** | **250M** | SE50 | | 12.5 | | ≥85% | | 4.5 | | 5.5 | |
|  |  |  | **SE75** | **18.5** | | **≥85%** | | **6.0** | | **7.0** | |  |
|  |  |  | PE75 | 37.5 | | ≥85% | | 11.5 | | 13.0 | |  |
|  |  |  | PE150 | 75.0 | | ≥80% | | 19.5 | | 21.0 | |  |

**Table S9. The pathogen detection range of tNGS platform**

| **Classification** | **Testing items** | | |
| --- | --- | --- | --- |
| **Gram positive bacteria (64 types)** | Corynebacterium diphtheriae | Mycobacterium kansasii | Enterococcus faecium |
|  | Mycobacterium intracellulare | Bacillus subtilis | Mycobacterium haemophilum |
|  | Staphylococcus epidermidis | Bacillus cereus | Bacillus anthracis |
|  | Mycobacterium bolletii | Staphylococcus lugdunensis | Nocardia terpenica |
|  | Mycobacterium xenopi | Rhodococcus hoagii | Streptococcus dysgalactiae |
|  | Clostridium perfringens | Mycobacterium malmoense | Nocardia otitidiscaviarum |
|  | Enterococcus gallinarum | Streptococcus milleri | Corynebacterium striatum |
|  | Listeria monocytogenes | Trueperella pyogenes | Staphylococcus warneri |
|  | **Streptococcus pneumoniae** | Streptococcus pyogenes | Streptococcus agalactiae |
|  | Enterococcus faecalis | Mycobacterium avium complex | Veillonella parvula |
|  | Nocardia cyriacigeorgica | Mycobacterium avium | Nocardia asteroides |
|  | Mycobacterium gordonae | Mycobacterium bovis | Streptococcus constellatus |
|  | Mycobacterium colombiense | Streptococcus bovis | Nocardia asiatica |
|  | Listeria grayi | Mycobacteroides abscessus | Peptostreptococcus anaerobius |
|  | Mycobacterium chelonae | Nocardia abscessus | Streptococcus anginosus |
|  | Mycobacterium marinum | Mycobacterium fortuitum | Enterococcus faecium |
|  | Streptococcus mitis | Nocardia farcinica | Actinomyces israelii |
|  | Tropheryma whipplei | Clostridium tetani | Mycobacterium simiae |
|  | Streptococcus cristatus | Mycobacterium tuberculosis | Streptococcus intermedius |
|  | Clostridioides difficile | Staphylococcus hominis | Streptococcus suis |
|  | Mycobacterium tuberculosis complex | Staphylococcus haemolyticus |  |
|  | **Staphylococcus aureus** | Clostridium botulinum |  |
| **Gram negative bacteria (60 types)** | Elizabethkingia anophelis | Bordetella parapertussis | Elizabethkingia meningoseptica |
|  | Bordetella pertussis | Haemophilus parainfluenzae | Neisseria meningitidis |
|  | Cronobacter sakazakii | Vibrio parahaemolyticus | Proteus vulgaris |
|  | **Acinetobacter baumannii** | Salmonella Paratyphi | Proteus mirabilis |
|  | Burkholderia mallei | Bartonella henselae | Cardiobacterium hominis |
|  | Citrobacter braakii | Elizabethkingia miricola | Legionella pneumophila |
|  | Pseudomonas alcaligenes | Vibrio cholerae | **Stenotrophomonas maltophilia** |
|  | Klebsiella aerogenes | Bordetella holmesii | Aeromonas hydrophila |
|  | Klebsiella oxytoca | Enterobacter hormaechei | Yersinia pestis |
|  | Chryseobacterium indologenes | Corynebacterium jeikeium | Burkholderia gladioli |
|  | Vibrio Vulnificus | Campylobacter rectus | **Pseudomonas aeruginosa** |
|  | Bacteroides fragilis | Kingella kingae | Parvimonas micra |
|  | Escherichia coli | Moraxella catarrhalis | Bartonella vinsonii |
|  | Pasteurella multocida | Citrobacter koseri | Yersinia enterocolitica |
|  | Burkholderia multivorans | Campylobacter jejuni | Porphyromonas gingivalis |
|  | Pseudomonas putida | Burkholderia pseudomallei | Burkholderia cepacia |
|  | **Klebsiella pneumoniae** | Neisseria gonorrhoeae | Serratia liquefaciens |
|  | Alcaligenes faecalis | **Haemophilus influenzae** | Acinetobacter calcoaceticus |
|  | Citrobacter freundii | Morganella morganii | Enterobacter cloacae |
| **DNA viruses (23 types)** | BK Polyomavirus | Human papillomavirus type 5 | Human herpesvirus 7 |
|  | EB virus | Human papillomavirus type 7 | Human herpesvirus 8 |
|  | JC Polyomavirus | Human herpesvirus type 1 | Varicella zoster virus |
|  | kI polyomavirus | Human herpesvirus type 2 | Torque teno virus |
|  | MW multi tumor virus | Human herpesvirus 6 | **Adenovirus** |
|  | TTV like parvovirus | Molluscum contagiosum virus | Adeno associated virus |
|  | WU polyomavirus | Monkeypox virus | Hepatitis B virus |
|  | **human bocavirus** | Cytomegalovirus |  |
| **RNA viruses (57 types)** | Echovirus | Yellow fever virus | Tick-borne encephalitis virus |
|  | Powassan virus | Poliovirus type 1 | Human rhinovirus |
|  | Enterovirus A | Poliovirus type 2 | Human coronavirus 229E |
|  | Enterovirus B | Poliovirus type 3 | Human coronavirus HKU1 |
|  | Enterovirus C | **Influenza A virus** | Human coronavirus NL63 |
|  | Enterovirus D | **Influenza B virus** | Human coronavirus OC43 |
|  | Dengue virus type 1 | Coxsackievirus A | human immunodeficiency virus 1 |
|  | Dengue virus type 2 | Coxsackievirus A16 | Primate T-lymphotropic virus 2 |
|  | Dengue virus type 3 | Coxsackievirus A21 | Primate T-lymphotropic virus 1 |
|  | Dengue virus type 4 | Coxsackievirus A6 | Human astrovirus |
|  | Eastern equine encephalitis virus | Coxsackievirus B | **Human metapneumovirus** |
|  | Rubella virus | Rabies virus | Tuscan virus |
|  | Parechovirus | La Crosse virus | West Nile virus |
|  | **Parainfluenza virus type 1: PIV1** | Lymphocytic choriomeningitis mammarenavirus | New Bunia virus |
|  | **Parainfluenza virus type 2: PIV2** | Mumps rubulavirus | Severe acute respiratory syndrome coronavirus 2 |
|  | **Parainfluenza virus type 3: PIV3** | Japanese encephalitis virus | Sapporo virus |
|  | **Parainfluenza virus type 4: PIV4** | Rotavirus | Zika virus |
|  | Hantavirus | Measles virus |  |
|  | **Respiratory syncytial virus A** | Encephalomyocarditis virus |  |
|  | **Respiratory syncytial virus B** | Norovirus |  |
| **Fungi (55 types)** | Trichosporon asahii | Cunninghamella bertholletiae | Exophiala dermatitidis |
|  | Paracoccidioides brasiliensis | Meyerozyma guilliermondii | Blastomyces dermatitidis |
|  | Geotrichum candidum | Histoplasma capsulatum | **Candida tropicalis** |
|  | **Candida albicans** | Fusarium oxysporum | Scedosporium |
|  | Cladophialophora bantiana | Scedosporium apiospermum | Lichtheimia corymbifera |
|  | Aspergillus clavatus | Wickerhamomyces anomalus | Sporothrix schenckii |
|  | Coccidioides posadasii | **Candida parapsilosis** | Magnusiomyces capitatus |
|  | Fusarium proliferatum | Mucor circinelloides | Aspergillus terreus |
|  | Fusarium verticillioides | Pichia kudriavzevii | Rhizomucor pusillus |
|  | Coccidioides immitis | Fusarium | Candida haemulonis |
|  | Candida dubliniensis | Schizophyllum commune | Rhizopus microsporus |
|  | Lomentospora prolificans | Clavispora lusitaniae | Cunninghamella |
|  | Cryptococcus gattii | Papiliotrema laurentii | Cryptococcus neoformans |
|  | Aspergillus nidulans | Talaromyces marneffei | Aspergillus fumigatus |
|  | **Nakaseomyces glabratus** | Mucor | Pneumocystis jirovecii |
|  | Fusarium graminearum | Rhizopus oryzae | Cladosporium cladosporioides |
|  | Aspergillus niger | Aspergillus oryzae | Mucor racemosus |
|  | Lichtheimia | Rhizomucor endophyticus |  |
|  | Aspergillus flavus | Saccharomyces cerevisiae |  |
| **atypical pathogen (29 types)** | Rickettsia typhi | Ureaplasma urealyticum | Paragonimus westermani |
|  | Coxiella burnetii | Rickettsia rickettsii | Leptospira interrogans |
|  | Orientia | Toxocara cati | Orientia tsutsugamushi |
|  | Plasmodium falciparum | Rickettsia felis | Trichomonas vaginalis |
|  | Chlamydia pneumoniae | Treponema pallidum | Cryptosporidium |
|  | **Mycoplasma pneumoniae** | Necator americanus | Chlamydia psittaci |
|  | Toxoplasma gondii | Taenia saginata | Taenia solium |
|  | Toxoplasma | Plasmodium | Chlamydia trachomatis |
|  | Angiostrongylus cantonensis | Mycoplasma hominis | Anaplasma phagocytophilum |
|  | Acanthamoeba | Entamoeba histolytica |  |
| **Genus (8 types)** | Brucella | Trichoderma | Cryptococcus |
|  | Rhizomucor sp | Stachybotrys | Bartonella |
|  | Alternaria | Salmonella |  |

**Table S10. Drug-resistance genes detected by tNGS in clinical samples and corresponding AST results**

| **Patient ID** | **Drug resistance genes detected by tNGS** | **Gender** | **Age** | **Antimicrobial Susceptibility Test** | |
| --- | --- | --- | --- | --- | --- |
|  |  |  |  | **Drug-resistance** | **Drug-sensitivity** |
| 3 | vanA | F | 61 | None (The cultivation result is fungal) | |
| 4 | ermB; tetM | F | 33 | None (The cultivation result is negative) | |
| 5 | tetM; ermB; TEM; tetL | F | 69 | None (The cultivation result is negative) | |
| 8 | ermB; tetL; TEM; tetM | F | 60 | None (The cultivation result is negative) | |
| 9 | tetQ; ermB; tetM | M | 16 | None (The cultivation result is negative) | |
| 11 | tetM; ermB | M | 74 | None (The cultivation result is negative) | |
| 12 | tetM; ermB; tetL | M | 63 | None (The cultivation result is negative) | |
| 17 | tetM; ermB | M | 75 | None (The cultivation result is fungal) | |
| 23 | TEM; tetM; ermB | M | 41 | None (The cultivation result is negative) | |
| 24 | OXA-1; TEM; OXA-23; KPC; CTX-M-1; AAC (6’); ADE; mefA; SHV; OXA-51; OXA-69; AAC (3’) | F | 58 | Ciprofloxacin, Levofloxacin, Moxifloxacin, Imipenem, Melopeinan, Cefazolin, Cefepime, Cefotaxime, Ceftazidime, Azertonam, Gentamicin, Amoxicillin/Clavulanic acid, Ampicillin/Sulbactam, Piperacillin/Tazobactam | Compound sulfamethoxazole, Tetracycline, Chloramphenicol, Amikacin |
| 25 | OXA-23; OXA-1; TEM; tetW; ADE; AAC (6’); mefA; floR; CTX-M-1 | M | 84 | Ciprofloxacin, Levofloxacin, Moxifloxacin, Compound sulfamethoxazole, Tetracycline, Cefazolin, Cefepime, Cefotaxime, Ceftazidime, Azertonam, Ampicillin/Sulbactam, Piperacillin/Tazobactam | Imipenem, Melopeinan, Gentamicin, Amikacin |
| 28 | OXA-23; msrA | M | 71 | None (The cultivation result is negative) | |
| 29 | OXA-23; ADE; | F | 63 | None (Discharge) | |
| 30 | APH; mecA; mefA; tetM | M | 84 | Ciprofloxacin, Levofloxacin, Moxifloxacin, Compound sulfamethoxazole, Tetracycline, Cefazolin, Cefepime, Cefotaxime, Ceftazidime, Azertonam, Ampicillin/Sulbactam, Piperacillin/Tazobactam | Imipenem, Melopeinan, Gentamicin, Amikacin |
| 32 | TEM | M | 72 | None (The cultivation result is negative) | |
| 33 | tetM; ermB | M | 74 | None (The cultivation result is negative) | |
| 34 | OXA-1; | M | 64 | Ciprofloxacin, Levofloxacin, Imipenem, Piperacillin, Cefepime, Ceftazidime, Azertonam, Piperacillin/Tazobactam | - |
| 35 | tetQ; OXA-1; TEM; CTX-M-1; AAC (6’); SHV; ermB | F | 78 | None (The cultivation result is negative) | |
| 36 | OXA-23; KPC; ADE; | M | 76 | Ciprofloxacin, Levofloxacin, Moxifloxacin, Compound sulfamethoxazole, Tetracycline, Ampicillin, Cefazolin, Cefepime, Cefotaxime, Ampicillin/Sulbactam, Chloramphenicol, Amikacin | Imipenem, Melopeinan, Ceftazidime, Amoxicillin/Clavulanic acid, Piperacillin/Tazobactam |
| 38 | tetM; TEM; ermB | F | 33 | None (The cultivation result is fungal) | |
| 39 | tetQ; tetM | M | 71 | None (The cultivation result is fungal) | |
| 40 | KPC; OXA-1; CTX-M-1; TEM; AAC (6’); SHV | M | 67 | Ciprofloxacin, Levofloxacin, Moxifloxacin, Cefazolin, Cefepime, Cefotaxime, Ceftazidime, Azertonam, Amoxicillin/Clavulanic acid, Ampicillin/Sulbactam, Piperacillin/Tazobactam, Gentamicin | Compound sulfamethoxazole, Tetracycline, Imipenem, Melopeinan, Chloramphenicol, Amikacin |
| 41 | KPC; SHV; OXA-23; | M | 93 | None (Discharge) | |
| 45 | tetM; ermB | F | 79 | - | Ciprofloxacin, Levofloxacin, Compound sulfamethoxazole, Imipenem, Melopeinan, Cefepime, Cefotaxime, Ceftazidime, Ampicillin/Sulbactam, Piperacillin/Tazobactam, Gentamicin, Amikacin |
| 46 | KPC; OXA-23; TEM; SHV; AAC (6’); ADE | F | 87 | Ciprofloxacin, Levofloxacin, Moxifloxacin, Imipenem, Melopeinan, Cefazolin, Cefepime, Cefotaxime, Ceftazidime, Azertonam, Amoxicillin/Clavulanic acid, Ampicillin/Sulbactam, Piperacillin/Tazobactam, Chloramphenicol, Amikacin | Compound sulfamethoxazole, Tetracycline |
| 47 | TEM | F | 79 | None (The cultivation result is negative) | |
| 48 | tetQ; tetM; ermB | M | 37 | None (The cultivation result is negative) | |
| 49 | tetW; OXA-23; KPC; TEM; OXA-1; vanA | M | 64 | Ciprofloxacin, Levofloxacin, Imipenem, Piperacillin, Cefepime, Ceftazidime, Azertonam, Piperacillin/Tazobactam | - |
| 50 | TEM; mefA; tetL | M | 65 | None (The cultivation result is fungal) | |
| 52 | OXA-23; TEM | F | 73 | Ciprofloxacin, Levofloxacin, Moxifloxacin, Compound sulfamethoxazole, Imipenem, Melopeinan, Cefazolin, Cefepime, Cefotaxime, Ceftazidime, Ampicillin/Sulbactam, Piperacillin/Tazobactam, Gentamicin | Amikacin |
| 53 | mefA | M | 83 | - | Ciprofloxacin, Levofloxacin, Compound sulfamethoxazole, Tetracycline, Imipenem, Melopeinan, Ceftazidime, Cefepime, Ampicillin/Sulbactam, Piperacillin/Tazobactam, |
| 55 | mefA | M | 66 | Cefepime, Ceftazidime, Azertonam | Ciprofloxacin, Levofloxacin, Imipenem, Melopeinan, Piperacillin, Piperacillin/Tazobactam, Amikacin |
| 56 | OXA-1; KPC; AAC (6'); CTX-M-1; TEM | M | 87 | Ciprofloxacin, Levofloxacin, Moxifloxacin, Imipenem, Melopeinan, Cefazolin, Cefepime, Cefotaxime, Ceftazidime, Azertonam, Amoxicillin/Clavulanic acid, Ampicillin/Sulbactam, Piperacillin/Tazobactam, Gentamicin | Compound sulfamethoxazole, Tetracycline, Chloramphenicol, Amikacin |
| 57 | mefA; mecA; tetW; OXA-23; TEM; APH | M | 87 | Ciprofloxacin, Levofloxacin, Compound sulfamethoxazole, Imipenem, Melopeinan, Ampicillin/Sulbactam, Piperacillin/Tazobactam, Gentamicin | Amikacin |
| 58 | AAC (6’); TEM; CTX-M-1; OXA-23; AAC (3’) | M | 76 | Ciprofloxacin, Levofloxacin, Imipenem, Melopeinan, Piperacillin, Ceftazidime, Cefepime, Azertonam, Piperacillin/Tazobactam, Amikacin | - |
| 59 | CTX-M-1 | M | 76 | Compound sulfamethoxazole, Tetracycline, Cefazolin, Cefepime, Cefotaxime, Ceftazidime, Azertonam | Ciprofloxacin, Levofloxacin, Moxifloxacin, Imipenem, Melopeinan, Amoxicillin/Clavulanic acid, Piperacillin/Tazobactam, Chloramphenicol, Amikacin |
| 60 | tetW; tetM; ermB | M | 73 | None (The cultivation result is fungal) | |
| 61 | NDM; TEM; AAC (6’); SHV; OXA-23; AAC (3’) | M | 75 | Ciprofloxacin, Levofloxacin, Compound sulfamethoxazole, Imipenem, Melopeinan, Cefazolin, Cefepime, Cefotaxime, Ceftazidime, Azertonam, Ampicillin/Sulbactam, Piperacillin/Tazobactam, Gentamicin, Amikacin | - |
| 62 | OXA-23; NDM; mefA; ADE; OXA-51; OXA-69; SHV; CAT; vanA; ARM | M | 61 | Ciprofloxacin, Levofloxacin, Compound sulfamethoxazole, Imipenem, Melopeinan, Cefepime, Cefotaxime, Ceftazidime, Ampicillin/Sulbactam, Piperacillin/Tazobactam, Gentamicin, Amikacin | - |
| 66 | OXA-1; KPC; AAC (6’); CTX-M-1; TEM | M | 87 | Ciprofloxacin, Levofloxacin, moxifloxacin, Imipenem, Melopeinan, Cefazolin, Cefepime, Cefotaxime, Ceftazidime, Azertonam, Amoxicillin/Clavulanic acid, Ampicillin/Sulbactam, Piperacillin/Tazobactam, Gentamicin | Compound sulfamethoxazole, Tetracycline, Chloramphenicol, Amikacin |
| 67 | tetQ; tetM; ermB; TEM | M | 18 | None (The cultivation result is negative) | |
| 69 | tetM; ermB | M | 63 | None (The cultivation result is negative) | |
| 71 | tetW; | F | 71 | - | Ciprofloxacin, Levofloxacin, Compound sulfamethoxazole, Tetracycline, Imipenem, Melopeinan, Cefepime, Cefotaxime, Ceftazidime, Ampicillin/Sulbactam, Piperacillin/Tazobactam, Amikacin |
| 72 | KPC; OXA-1; CTX-M-1 | F | 66 | Ciprofloxacin, Levofloxacin, moxifloxacin, Imipenem, Melopeinan, Cefazolin, Cefepime, Cefotaxime, Ceftazidime, Azertonam, Amoxicillin/Clavulanic acid, Ampicillin/Sulbactam, Piperacillin/Tazobactam, Cefotaxime/Avibactam, Gentamicin | Compound sulfamethoxazole, Tetracycline, Chloramphenicol, Amikacin |
| 73 | OXA-1; KPC | F | 66 | None (Discharge) | |
| 75 | tetW; mefA; mecA; OXA-23; TEM; APH; QAC | F | 69 | Ciprofloxacin, Levofloxacin, moxifloxacin, Imipenem, Melopeinan, Cefazolin, Cefepime, Cefotaxime, Ceftazidime, Azertonam, Amoxicillin/Clavulanic acid, Ampicillin/Sulbactam, Piperacillin/Tazobactam, Chloramphenicol | Compound sulfamethoxazole, Tetracycline, Amikacin |
| 76 | mecA | M | 89 | - | Ciprofloxacin, Levofloxacin, Compound sulfamethoxazole, Imipenem, Melopeinan, Cefepime, Cefotaxime, Ceftazidime, Azertonam, Ampicillin/Sulbactam, Piperacillin/Tazobactam, Gentamicin, Amikacin |
| 79 | KPC; OXA-1; CTX-M-1; TEM; AAC (6’); OXA-23 | F | 69 | Ciprofloxacin, Levofloxacin, moxifloxacin, Imipenem, Melopeinan, Cefazolin, Cefepime, Cefotaxime, Ceftazidime, Azertonam, Amoxicillin/Clavulanic acid, Ampicillin/Sulbactam, Piperacillin/Tazobactam, Chloramphenicol | Compound sulfamethoxazole, Tetracycline, Amikacin |
| 80 | tetM; ermB | F | 62 | None (Discharge) | |
| 81 | tetL; tetM; ermB | F | 48 | Ciprofloxacin, Tetracycline, Cefepime, Cefotaxime, Ceftazidime, Azertonam, | Compound sulfamethoxazole, Imipenem, Gentamicin, Amikacin |
| 83 | tetQ; OXA-23; tetW; TEM | M | 79 | None (The cultivation result is negative) | |
| 84 | tetQ; ermB | M | 25 | None (Discharge) | |
| 87 | OXA-23; AAC (6’); TEM | M | 92 | Ciprofloxacin, Levofloxacin, Compound sulfamethoxazole, Imipenem, Melopeinan, Cefepime, Cefotaxime, Ceftazidime, Ampicillin/Sulbactam, Piperacillin/Tazobactam, Gentamicin, Amikacin | - |
| 88 | TEM; ermB; tetM; mefA | M | 79 | - | Ciprofloxacin, Levofloxacin, moxifloxacin, Compound sulfamethoxazole, Tetracycline, Imipenem, Melopeinan, Cefazolin, Cefepime, Cefotaxime, Ceftazidime, Azertonam, Amoxicillin/Clavulanic acid, Ampicillin/Sulbactam, Piperacillin/Tazobactam, Chloramphenicol, Amikacin |
| 89 | KPC; TEM; SHV | M | 87 | Ciprofloxacin, Levofloxacin, moxifloxacin, Imipenem, Melopeinan, Cefazolin, Cefepime, Cefotaxime, Ceftazidime, Azertonam, Amoxicillin/Clavulanic acid, Ampicillin/Sulbactam, Piperacillin/Tazobactam, Gentamicin, Amikacin | Compound sulfamethoxazole, Tetracycline, Chloramphenicol |
| 91 | tetW; mecA mefA; APH; tetM; QAC; vanA | M | 84 | Ciprofloxacin, Levofloxacin, Imipenem, Melopeinan, piperacillin, Cefepime, Ceftazidime, Azertonam, Piperacillin/Tazobactam | Amikacin |
| 92 | tetW; tetM; ermB | M | 81 | None (Discharge) | |
| 93 | mefA; QAC | M | 66 | - | Ciprofloxacin, Levofloxacin, Compound sulfamethoxazole, Imipenem, Melopeinan, Cefepime, Cefotaxime, Ceftazidime, Azertonam, Amoxicillin/Clavulanic acid, Ampicillin/Sulbactam, Piperacillin/Tazobactam, Chloramphenicol, Gentamicin, Amikacin |
| 94 | OXA-1; cmlA; floR; AAC (6’); CMY; KPC; AAC (3’); CTX-M-1 | F | 60 | Imipenem | Piperacillin, Ceftazidime, Cefepime, Piperacillin/Tazobactam, Amikacin |
| 96 | OXA-1; OXA-23; AAC (6’); AAC (3’) | M | 64 | Ciprofloxacin, Levofloxacin, piperacillin, Cefepime, Ceftazidime, Azertonam, Piperacillin/Tazobactam, Gentamicin, | Imipenem, Melopeinan, Amikacin |

Table S11. Resistance genes and its related drugs

| **Resistance genes** | **Drugs** |
| --- | --- |
| TEM | Penicillin, First generation cephalosporin, Second-generation cephalosporin, Imipenem, Meropenem, Ampicillin |
| mefA | Erythromycin, Azithromycin, Clarithromycin |
| tetL | Tetracycline, OxyTetracycline, Doxycycline, Minocycline |
| tetQ | Tetracycline, Doxycycline, OxyTetracycline |
| tetM | Tetracycline, OxyTetracycline, Doxycycline, Minocycline |
| ermB | Erythromycin, Azithromycin, Clarithromycin, Lincomycin |
| OXA-23 | Meropenem, Imipenem, Penicillin, Cephalosporins, Aztreonam |
| mecA | Methicillin, Carbapenems, Penicillin, Cephalosporins |
| QAC | Disinfectant resistant gene |
| tetW | Tetracycline, Doxycycline, Minocycline |
| CTX-M-1 | Penicillin, Ampicillin, Amoxicillin, First generation cephalosporin, Second-generation cephalosporin, Third generation cephalosporin, Aztreonam |
| floR | Norfloxacin, Levofloxacin, ciprofloxacin |
| AAC (6’) | Streptomycin, Gentamicin, Kanamycin, Tobramycin, Amikacin |
| KPC | Meropenem, Imipenem, Doripenem, Etapenem, Penicillins, Cephalosporins |
| SHV | Penicillin G, Ampicillin, Ceftriaxone, Ceftriaxone, Cefepime, Imipenem, Meropenem |
| APH | Gentamicin, Kanamycin, Amikacin, Neomycin, Tobramycin, Streptomycin |
| vanA | Vancomycin, Teicoplanin, Carbapenem |
| ADE | Exogenous pump gene |
| OXA-51 | Cefotaxime, Cefotaxime, Imipenem, Penicillins G, Ampicillin |
| OXA-69 | Cephalosporins, Imipenem, Meropenem, Penicillin G, Ampicillin, Aztreonam |
| OXA-1 | Penicillin G, Ampicillin, Amoxicillin, Cephalothin, Cefazolin, Ceftriaxone |
| cmlA | Chloramphenicol |
| CMY | Ampicillin, Amoxicillin, First generation cephalosporin, Second-generation cephalosporin, Meropenem, Imipenem, Aztreonam |
| AAC (3’) | Streptomycin, Gentamicin, Kanamycin, Tobramycin, Amikacin |
| NDM | Ppenicillin G, Ampicillin, Amoxicillin, Cephalothin, Cefazolin, Ceftriaxone, Imipenem, Meropenem, Aztreonam |
| CAT | Chloramphenicol |
| ARM | Gentamicin, Kanamycin, Amikacin, Neomycin, Tobramycin, Streptomycin |
| msrA | Tetracycline, Doxycycline, Minocycline, Erythromycin |

Table S12. 97 positive clinical samples detected by tNGS

|  | **Organism** | **Reads count** | **Correctly matched reads** | **RPM** | **Gender** | **Age** |
| --- | --- | --- | --- | --- | --- | --- |
| 1 | C. albicans | 21711 | 921725 | 23554.75 | F | 79 |
|  | S. maltophilia | 71 | 921725 | 77.03 |  |  |
| 2 | C. albicans | 138604 | 1979621 | 70015.42 | F | 73 |
| 3 | L.corynebacterium | 55641 | 1403317 | 39649.63 | F | 61 |
| 4 | S. pneumoniae | 100 | 1256471 | 79.59 | F | 33 |
|  | H.influenzae | 76 | 1256471 | 60.49 |  |  |
| 5 | H.influenzae | 11534 | 333357 | 34599.54 | F | 69 |
|  | Nocardia genus | 1301 | 333357 | 3902.722907 |  |  |
|  | S. pneumoniae | 1222 | 333357 | 3665.739732 |  |  |
| 6 | M.pneumoniae | 45928 | 1042985 | 44035.15 | F | 37 |
| 7 | H.influenzae | 156 | 843354 | 184.98 | M | 70 |
| 8 | S. pneumoniae | 4237 | 721757 | 5870.40 | F | 60 |
|  | H.influenzae | 776 | 721757 | 1075.154103 |  |  |
| 9 | S. pneumoniae | 7431 | 1085866 | 6843.385832 | M | 16 |
|  | S. aureus | 1249 | 1085866 | 1150.234007 |  |  |
|  | C.pneumoniae | 73 | 1085866 | 67.23 |  |  |
|  | C. parapsilosi | 112 | 1085866 | 103.1434818 |  |  |
| 10 | C. albicans | 12147 | 1321976 | 9188.52 | M | 71 |
|  | H.influenzae | 4732 | 1321976 | 3579.490097 |  |  |
|  | S. pneumoniae | 1655 | 1321976 | 1251.913802 |  |  |
| 11 | S. pneumoniae | 718 | 492211 | 1458.72 | M | 74 |
| 12 | H.influenzae | 5294 | 1499979 | 3529.382745 | M | 63 |
|  | S. pneumoniae | 1742 | 1499979 | 1161.349592 |  |  |
| 13 | S. pneumoniae | 247 | 381351 | 647.6972658 | F | 36 |
| 14 | H.influenzae | 113411 | 1773897 | 63933.24979 | F | 61 |
|  | A. fumigatus | 5 | 1773897 | 2.818652943 |  |  |
|  | Cytomegalovirus | 8014 | 1773897 | 4517.74 |  |  |
| 15 | H.influenzae | 4760 | 487386 | 9766.386396 | F | 41 |
|  | M.pneumoniae | 188 | 487386 | 385.73 |  |  |
| 16 | C. albicans | 1386 | 1145706 | 1209.73 | M | 71 |
|  | H.influenzae | 616 | 1145706 | 537.6597487 |  |  |
|  | G. capitatum | 57 | 1145706 | 49.75098324 |  |  |
| 17 | C. albicans | 13830 | 1309627 | 10560.26 | M | 75 |
|  | Cytomegalovirus | 629 | 1309627 | 480.29 |  |  |
|  | M. catarrhalis | 121 | 1309627 | 92.39 |  |  |
|  | P. jiroveci | 111 | 1309627 | 84.76 |  |  |
| 18 | A. baumanii | 191 | 738124 | 258.76 | M | 76 |
| 19 | A. fumigatus | 8988 | 343848 | 26139.46 | M | 87 |
|  | S. marcescens | 25 | 343848 | 72.71 |  |  |
|  | A. baumanii | 53 | 343848 | 154.1378749 |  |  |
| 20 | C. albicans | 132155 | 1166197 | 113321.33 | F | 63 |
| 21 | C.neoformans | 266 | 429647 | 619.11 | F | 71 |
|  | S. aureus | 60 | 429647 | 139.6495262 |  |  |
|  | A. baumanii | 17 | 429647 | 39.56736577 |  |  |
| 22 | C. albicans | 109813 | 1516968.00 | 72389.79 | F | 87 |
|  | S. pneumoniae | 373 | 1516968.00 | 245.89 |  |  |
|  | Candida glabrata | 9098 | 1516968.00 | 5997.49 |  |  |
| 23 | H.influenzae | 68768 | 1468629 | 46824.62 | M | 41 |
|  | S. pneumoniae | 503 | 1468629 | 342.496301 |  |  |
| 24 | A. baumanii | 19556 | 1044754 | 18718.28 | F | 58 |
|  | K. pneumoniae | 13890 | 1044754 | 13295.00 |  |  |
|  | P.aeruginosa | 4293 | 1044754 | 4109.10 |  |  |
|  | C. albicans | 88 | 1044754 | 84.23 |  |  |
| 25 | A. baumanii | 32280 | 1055005 | 30597.01 | M | 84 |
|  | K. pneumoniae | 3069 | 1055005 | 2908.99 |  |  |
|  | C. striatum | 1667 | 1055005 | 1580.09 |  |  |
|  | P.aeruginosa | 995 | 1055005 | 943.12 |  |  |
|  | C. parapsilosi | 439 | 1055005 | 416.11 |  |  |
|  | M. morganii | 353 | 1055005 | 334.60 |  |  |
|  | S. aureus | 264 | 1055005 | 250.2357809 |  |  |
|  | S. maltophilia | 159 | 1055005 | 150.7101862 |  |  |
|  | E. meningoseptica | 2880 | 1055005 | 2729.844882 |  |  |
|  | Escherichia coli | 34 | 1055005 | 32.22733542 |  |  |
|  | Candida glabrata | 6 | 1055005 | 5.687176838 |  |  |
| 26 | Cytomegalovirus | 11396 | 759205 | 15010.44 | F | 74 |
|  | P. jiroveci | 1916 | 759205 | 2523.69 |  |  |
|  | S. pneumoniae | 158 | 759205 | 208.1124334 |  |  |
|  | M. catarrhalis | 1371 | 759205 | 1805.84 |  |  |
|  | C. albicans | 1338 | 759205 | 1762.37 |  |  |
| 27 | H.influenzae | 11253 | 1396468 | 8058.19 | F | 68 |
|  | C. albicans | 196 | 1396468 | 140.35 |  |  |
| 28 | A. baumanii | 14109 | 485073 | 29086.34 | M | 71 |
|  | H.influenzae | 1127 | 485073 | 2323.36 |  |  |
|  | P.aeruginosa | 1095 | 485073 | 2257.392186 |  |  |
|  | S. aureus | 63 | 485073 | 129.8773587 |  |  |
|  | S. maltophilia | 656 | 485073 | 1352.373766 |  |  |
|  | adv | 126 | 485073 | 259.75 |  |  |
| 29 | A. baumanii | 52317 | 1551858 | 33712.49 | F | 63 |
|  | P.aeruginosa | 17348 | 1551858 | 11178.86 |  |  |
|  | S. maltophilia | 15350 | 1551858 | 9891.37 |  |  |
| 30 | S. aureus | 32051 | 1339060 | 23935.45 | M | 84 |
|  | C. striatum | 18763 | 1339060 | 14012.07 |  |  |
|  | S. maltophilia | 13716 | 1339060 | 10243.01 |  |  |
|  | E. meningoseptica | 1610 | 1339060 | 1202.335967 |  |  |
|  | C. parapsilosi | 76 | 1339060 | 56.75623198 |  |  |
|  | P.aeruginosa | 7089 | 1339060 | 5294.01 |  |  |
|  | K. pneumoniae | 60 | 1339060 | 44.81 |  |  |
| 31 | H.influenzae | 70823 | 2433873 | 29098.89 | M | 63 |
|  | C. albicans | 264 | 2433873 | 108.4690943 |  |  |
|  | adv | 159 | 2433873 | 65.32797726 |  |  |
|  | Human herpesvirus 1 | 416 | 2433873 | 170.92 |  |  |
| 32 | H.influenzae | 15851 | 1359986 | 11655.27 | M | 72 |
| 33 | H.influenzae | 649 | 2486143 | 261.05 | M | 74 |
|  | S. pneumoniae | 209 | 2486143 | 84.07 |  |  |
|  | K. pneumoniae | 64 | 2486143 | 25.74 |  |  |
| 34 | P.aeruginosa | 36005 | 1122649 | 32071.47 | M | 64 |
|  | A. baumanii | 5564 | 1122649 | 4956.13 |  |  |
|  | S. maltophilia | 670 | 1122649 | 596.80 |  |  |
|  | K. pneumoniae | 430 | 1122649 | 383.02 |  |  |
|  | S. aureus | 66 | 1122649 | 58.79 |  |  |
| 35 | K. pneumoniae | 39912 | 1363151 | 29279.22 | F | 78 |
|  | S. aureus | 1272 | 1363151 | 933.13 |  |  |
| 36 | A. baumanii | 92835 | 1487827 | 62396.37 | M | 76 |
|  | P.aeruginosa | 1943 | 1487827 | 1305.93 |  |  |
|  | K. pneumoniae | 1902 | 1487827 | 1278.37 |  |  |
|  | Escherichia coli | 985 | 1487827 | 662.04 |  |  |
|  | S. maltophilia | 110 | 1487827 | 73.93 |  |  |
| 37 | S. maltophilia | 41488 | 1064605 | 38970.32 | F | 63 |
|  | Human herpesvirus 1 | 25103 | 1064605 | 23579.64 |  |  |
|  | P.aeruginosa | 8 | 1064605 | 7.51 |  |  |
| 38 | C. albicans | 8232 | 950982 | 8656.32 | F | 33 |
|  | H.influenzae | 7787 | 950982 | 8188.377908 |  |  |
|  | Mucor | 3008 | 950982 | 3163.046199 |  |  |
|  | A. baumanii | 1412 | 950982 | 1484.78 |  |  |
| 39 | C. albicans | 22319 | 946104 | 23590.43 | M | 71 |
|  | Candida glabrata | 237 | 946104 | 250.501002 |  |  |
|  | S. aureus | 95 | 946104 | 100.4117941 |  |  |
|  | K. pneumoniae | 1186 | 946104 | 1253.56 |  |  |
|  | Influenza virus A | 373 | 946104 | 394.25 |  |  |
| 40 | K. pneumoniae | 55387 | 1458798 | 37967.56 | M | 67 |
|  | S. maltophilia | 17880 | 1458798 | 12256.67 |  |  |
|  | C. albicans | 160 | 1458798 | 109.68 |  |  |
| 41 | K. pneumoniae | 22439 | 841099 | 26678.19 | M | 93 |
|  | A. baumanii | 18361 | 841099 | 21829.77 |  |  |
|  | S. aureus | 8635 | 841099 | 10266.33 |  |  |
|  | Human herpesvirus 1 | 4615 | 841099 | 5486.87 |  |  |
|  | S. maltophilia | 54 | 841099 | 64.20 |  |  |
| 42 | C. albicans | 5533 | 1427813 | 3875.16 | M | 75 |
|  | P. putida | 16 | 1427813 | 11.20594924 |  |  |
| 43 | P.aeruginosa | 13687 | 492004 | 27818.88 | M | 66 |
|  | Nocardia genus | 3038 | 492004 | 6174.746547 |  |  |
|  | Aspergillus genus | 171 | 492004 | 347.5581499 |  |  |
|  | Cytomegalovirus | 67 | 492004 | 136.1777547 |  |  |
| 44 | M.pneumoniae | 35490 | 1143800 | 31028.15 | M | 14 |
|  | S. pneumoniae | 208 | 1143800 | 181.8499738 |  |  |
|  | H.influenzae | 97 | 1143800 | 84.80503585 |  |  |
|  | S. maltophilia | 10 | 1143800 | 8.74 |  |  |
|  | C. albicans | 805 | 1143800 | 703.79 |  |  |
| 45 | H.influenzae | 23759 | 1271575 | 18684.70 | F | 79 |
|  | A. baumanii | 3767 | 1271575 | 2962.47 |  |  |
|  | adv | 3249 | 1271575 | 2555.10 |  |  |
|  | K. pneumoniae | 27 | 1271575 | 21.23350962 |  |  |
|  | C. albicans | 307 | 1271575 | 241.43 |  |  |
| 46 | A. baumanii | 47544 | 1194879 | 39789.80 | F | 87 |
|  | K. pneumoniae | 20543 | 1194879 | 17192.54 |  |  |
|  | P.aeruginosa | 885 | 1194879 | 740.66 |  |  |
|  | C. albicans | 114 | 1194879 | 95.41 |  |  |
| 47 | H.influenzae | 16028 | 616505 | 25998.17 | F | 79 |
|  | C. albicans | 3926 | 616505 | 6368.16 |  |  |
|  | P.aeruginosa | 143 | 616505 | 231.95 |  |  |
| 48 | H.influenzae | 114620 | 1378007 | 83178.10 | M | 37 |
|  | S. aureus | 730 | 1378007 | 529.75 |  |  |
| 49 | A. baumanii | 24055 | 1414344 | 17007.88 | M | 64 |
|  | K. pneumoniae | 14370 | 1414344 | 10160.19 |  |  |
|  | Human herpesvirus 1 | 1262 | 1414344 | 892.29 |  |  |
|  | P.aeruginosa | 569 | 1414344 | 402.31 |  |  |
|  | S. maltophilia | 134 | 1414344 | 94.74 |  |  |
| 50 | H.influenzae | 35277 | 762292 | 46277.54 | M | 65 |
|  | C. albicans | 34272 | 762292 | 44959.15 |  |  |
|  | Human herpesvirus 1 | 142 | 762292 | 186.28 |  |  |
|  | Human herpesvirus 7 | 604 | 762292 | 792.3472895 |  |  |
|  | T. whipplei | 152 | 762292 | 199.3986556 |  |  |
| 51 | C. albicans | 168342 | 1051699 | 160066.71 | F | 78 |
| 52 | A. baumanii | 278504 | 1435478 | 194014.82 | F | 73 |
| 53 | S. maltophilia | 70985 | 850096 | 83502.33385 | M | 83 |
|  | Human herpesvirus 1 | 32590 | 850096 | 38336.84666 |  |  |
|  | C. tropicalis | 5195 | 850096 | 6111.07 |  |  |
|  | EB virus | 4999 | 850096 | 5880.51 |  |  |
|  | C. albicans | 87 | 850096 | 102.34 |  |  |
| 54 | P.aeruginosa | 100324 | 1099808 | 91219.56 | M | 68 |
|  | A. baumanii | 2468 | 1099808 | 2244.03 |  |  |
|  | S. aureus | 51 | 1099808 | 46.37173034 |  |  |
|  | Cytomegalovirus | 68 | 1099808 | 61.83 |  |  |
| 55 | P.aeruginosa | 45876 | 781617 | 58693.71 | M | 66 |
|  | Candida glabrata | 1133 | 781617 | 1449.56 |  |  |
|  | EB virus | 264 | 781617 | 337.76 |  |  |
| 56 | K. pneumoniae | 63800 | 686758 | 92900.26 | M | 87 |
|  | S. aureus | 202 | 686758 | 294.1356344 |  |  |
|  | C. tropicalis | 101 | 686758 | 147.0678172 |  |  |
| 57 | S. aureus | 103308 | 1087284 | 95014.73 | M | 87 |
|  | A. baumanii | 83036 | 1087284 | 76370.11 |  |  |
|  | K. pneumoniae | 4702 | 1087284 | 4324.54 |  |  |
|  | S. maltophilia | 1043 | 1087284 | 959.2709908 |  |  |
|  | C. albicans | 818 | 1087284 | 752.33 |  |  |
| 58 | A. baumanii | 76549 | 824475 | 92845.75 | M | 76 |
|  | K. pneumoniae | 54651 | 824475 | 66285.82 |  |  |
|  | P.aeruginosa | 879 | 824475 | 1066.13 |  |  |
|  | Escherichia coli | 1200 | 824475 | 1455.471664 |  |  |
|  | C. tropicalis | 115 | 824475 | 139.48 |  |  |
|  | C. albicans | 103 | 824475 | 124.93 |  |  |
| 59 | C. albicans | 96090 | 1408859 | 68204.13 | M | 76 |
|  | K. pneumoniae | 66673 | 1408859 | 47324.11 |  |  |
|  | Candida glabrata | 18056 | 1408859 | 12816.04 |  |  |
|  | Escherichia coli | 545 | 1408859 | 386.8378596 |  |  |
|  | E. hormaechei | 141 | 1408859 | 100.0809875 |  |  |
|  | C. parapsilosi | 51 | 1408859 | 36.19950613 |  |  |
|  | C. tropicalis | 16014 | 1408859 | 11366.64 |  |  |
|  | A. baumanii | 14840 | 1408859 | 10533.35 |  |  |
| 60 | S. maltophilia | 49266 | 1641374 | 30015.10 | M | 73 |
|  | C. albicans | 43720 | 1641374 | 26636.22 |  |  |
|  | EB virus | 24893 | 1641374 | 15165.95 |  |  |
|  | Human polyoma virus 5 | 282 | 1641374 | 171.81 |  |  |
|  | Human herpesvirus 7 | 1566 | 1641374 | 954.078717 |  |  |
|  | Human herpesvirus 1 | 81 | 1641374 | 49.35 |  |  |
|  | Cytomegalovirus | 79 | 1641374 | 48.13 |  |  |
| 61 | A. baumanii | 80608 | 1283036 | 62825.98 | M | 75 |
|  | K. pneumoniae | 44112 | 1283036 | 34380.95 |  |  |
|  | P.aeruginosa | 1768 | 1283036 | 1377.98 |  |  |
|  | S. maltophilia | 119 | 1283036 | 92.75 |  |  |
| 62 | A. baumanii | 65555 | 1541261 | 42533.35 | M | 61 |
|  | K. pneumoniae | 10058 | 1541261 | 6525.83 |  |  |
|  | S. maltophilia | 2319 | 1541261 | 1504.61 |  |  |
|  | Candida glabrata | 67 | 1541261 | 43.47 |  |  |
| 63 | P.aeruginosa | 86985 | 1114121 | 78075.00 | M | 66 |
| 64 | S. maltophilia | 51463 | 756367 | 68039.72 | F | 98 |
|  | P.aeruginosa | 41074 | 756367 | 54304.33 |  |  |
|  | K. pneumoniae | 93 | 756367 | 122.956184 |  |  |
|  | Human herpesvirus 1 | 19543 | 756367 | 25837.99 |  |  |
| 65 | M.pneumoniae | 30618 | 1185213 | 25833.33 | F | 34 |
|  | K. pneumoniae | 81 | 1185213 | 68.34 |  |  |
|  | A. baumanii | 258 | 1185213 | 217.6823913 |  |  |
|  | S. aureus | 158 | 1185213 | 133.3093714 |  |  |
| 66 | K. pneumoniae | 110333 | 1907078 | 57854.47685 | M | 87 |
|  | C. tropicalis | 69 | 1907078 | 36.18100571 |  |  |
| 67 | H.influenzae | 9931 | 1154205 | 8604.19 | M | 18 |
|  | adv group C | 8005 | 1154205 | 6935.51 |  |  |
|  | adv | 5314 | 1154205 | 4604.03 |  |  |
|  | adv 1 | 4848 | 1154205 | 4200.29 |  |  |
|  | A. baumanii | 163 | 1154205 | 141.2227464 |  |  |
|  | S. aureus | 112 | 1154205 | 97.03648832 |  |  |
|  | EB virus | 1245 | 1154205 | 1078.66 |  |  |
| 68 | H.influenzae | 47575 | 1758209 | 27058.79 | M | 69 |
| 69 | S. pneumoniae | 662 | 2036704 | 325.03 | M | 63 |
| 70 | P.aeruginosa | 8924 | 496406 | 17977.22 | M | 87 |
|  | C. albicans | 583 | 496406 | 1174.441888 |  |  |
|  | S. aureus | 155 | 496406 | 312.2444128 |  |  |
|  | C. parapsilosi | 87 | 496406 | 175.2597672 |  |  |
|  | Cytomegalovirus | 73 | 496406 | 147.06 |  |  |
| 71 | A. baumanii | 33216 | 1472986 | 22550.11 | F | 71 |
|  | K. pneumoniae | 496 | 1472986 | 336.73 |  |  |
|  | S. maltophilia | 137 | 1472986 | 93.01 |  |  |
|  | K. oxytoca | 13 | 1472986 | 8.83 |  |  |
| 72 | K. pneumoniae | 76524 | 905656 | 84495.66 | F | 66 |
|  | S. maltophilia | 5 | 905656 | 5.520860017 |  |  |
|  | S. aureus | 159 | 905656 | 175.56 |  |  |
| 73 | Human herpesvirus 1 | 147156 | 2522042 | 58347.96 | F | 66 |
|  | K. pneumoniae | 32201 | 2522042 | 12767.83 |  |  |
| 74 | Candida glabrata | 85615 | 2580516 | 33177.47 | M | 85 |
|  | C. albicans | 30447 | 2580516 | 11798.80 |  |  |
|  | A. baumanii | 21793 | 2580516 | 8445.21 |  |  |
|  | K. pneumoniae | 15991 | 2580516 | 6196.82 |  |  |
| 75 | A. baumanii | 88825 | 2257088 | 39353.80 | F | 69 |
|  | S. aureus | 88090 | 2257088 | 39028.16 |  |  |
|  | K. pneumoniae | 2316 | 2257088 | 1026.10 |  |  |
|  | C. albicans | 325 | 2257088 | 143.99 |  |  |
| 76 | C. albicans | 80052 | 1466189 | 54598.69 | M | 89 |
|  | Human herpesvirus 1 | 12311 | 1466189 | 8396.60 |  |  |
|  | A. baumanii | 1732 | 1466189 | 1181.29 |  |  |
|  | S. aureus | 57 | 1466189 | 38.87629767 |  |  |
| 77 | P.aeruginosa | 52434 | 10940067 | 4792.84 | M | 64 |
|  | S. maltophilia | 29082 | 10940067 | 2658.30 |  |  |
|  | K. pneumoniae | 14261 | 10940067 | 1303.56 |  |  |
|  | E. meningoseptica | 377 | 10940067 | 34.46048365 |  |  |
|  | EB virus | 57 | 10940067 | 5.210205751 |  |  |
|  | C. albicans | 644 | 10940067 | 58.87 |  |  |
| 78 | P.aeruginosa | 40445 | 1666945 | 24262.95 | F | 65 |
|  | H.influenzae | 18268 | 1666945 | 10958.97 |  |  |
| 79 | K. pneumoniae | 84026 | 1619581 | 51881.32 | F | 69 |
|  | A. baumanii | 49493 | 1619581 | 30559.14 |  |  |
|  | S. aureus | 3875 | 1619581 | 2392.59 |  |  |
|  | C. albicans | 106 | 1619581 | 65.45 |  |  |
| 80 | EB virus | 555 | 1466199 | 378.53 | F | 62 |
|  | S. pneumoniae | 351 | 1466199 | 239.39 |  |  |
|  | H.influenzae | 81 | 1466199 | 55.24 |  |  |
| 81 | C. albicans | 306 | 1471075 | 208.01 | F | 48 |
|  | A. baumanii | 127 | 1471075 | 86.33 |  |  |
|  | M.pneumoniae | 9 | 1471075 | 6.11797495 |  |  |
|  | K. pneumoniae | 67 | 1471075 | 45.54 |  |  |
| 82 | S. aureus | 423 | 131464 | 3217.61 | M | 60 |
|  | A. baumanii | 75 | 131464 | 570.4983874 |  |  |
| 83 | A. baumanii | 92597 | 486050 | 190509.21 | M | 79 |
|  | P.aeruginosa | 24958 | 486050 | 51348.63 |  |  |
|  | K. pneumoniae | 3421 | 486050 | 7038.370538 |  |  |
|  | EB virus | 876 | 486050 | 1802.283716 |  |  |
|  | Human polyoma virus 5 | 34 | 486050 | 69.95165106 |  |  |
|  | C. albicans | 665 | 486050 | 1368.171999 |  |  |
|  | S. aureus | 330 | 486050 | 678.9424956 |  |  |
|  | Escherichia coli | 241 | 486050 | 495.833762 |  |  |
|  | H.influenzae | 210 | 486050 | 432.0543154 |  |  |
|  | K. oxytoca | 26 | 486050 | 53.49243905 |  |  |
|  | Human herpesvirus 1 | 197 | 486050 | 405.31 |  |  |
| 84 | S. aureus | 446 | 611817 | 728.98 | M | 25 |
| 85 | A. baumanii | 678 | 444157 | 1526.487256 | M | 32 |
|  | S. aureus | 569 | 444157 | 1281.078538 |  |  |
|  | E. hormaechei | 14 | 444157 | 31.52038581 |  |  |
|  | P.aeruginosa | 228 | 444157 | 513.3319975 |  |  |
| 86 | S. aureus | 698 | 330962 | 2109.003451 | M | 65 |
| 87 | A. baumanii | 192488 | 1041046 | 184898.65 | M | 92 |
|  | S. maltophilia | 9507 | 1041046 | 9132.16 |  |  |
|  | P.aeruginosa | 6301 | 1041046 | 6052.57 |  |  |
|  | K. pneumoniae | 716 | 1041046 | 687.77 |  |  |
|  | L. pneumophila | 294 | 1041046 | 282.41 |  |  |
| 88 | C. albicans | 1276 | 821186 | 1553.85 | M | 79 |
|  | EB virus | 68155 | 821186 | 82995.81 |  |  |
|  | K. pneumoniae | 266 | 821186 | 323.92 |  |  |
| 89 | K. pneumoniae | 73581 | 681151 | 108024.51 | M | 87 |
|  | S. aureus | 2001 | 681151 | 2937.67 |  |  |
|  | Escherichia coli | 333 | 681151 | 488.88 |  |  |
| 90 | S. maltophilia | 61303 | 722481 | 84850.67427 | M | 86 |
|  | P.aeruginosa | 39399 | 722481 | 54532.92197 |  |  |
|  | C. albicans | 221 | 722481 | 305.8903971 |  |  |
|  | C. tropicalis | 9 | 722481 | 12.457075 |  |  |
| 91 | S. aureus | 165325 | 1033654 | 159942.3018 | M | 84 |
|  | C. tropicalis | 159 | 1033654 | 153.8232329 |  |  |
|  | K. pneumoniae | 74 | 1033654 | 71.59068702 |  |  |
|  | E. meningoseptica | 16 | 1033654 | 15.47906746 |  |  |
|  | Human herpesvirus 1 | 61 | 1033654 | 59.0139447 |  |  |
| 92 | C. krusei | 11945 | 1051349 | 11361.59353 | M | 81 |
|  | A. baumanii | 218 | 1051349 | 207.3526488 |  |  |
|  | K. pneumoniae | 179 | 1051349 | 170.2574502 |  |  |
| 93 | C. albicans | 48331 | 1103569 | 43795.17728 | M | 66 |
|  | EB virus | 876 | 1103569 | 793.79 |  |  |
|  | K. pneumoniae | 692 | 1103569 | 627.06 |  |  |
|  | Human herpesvirus 1 | 521 | 1103569 | 472.10 |  |  |
| 94 | P.aeruginosa | 62480 | 700630 | 89176.88 | F | 60 |
|  | K. pneumoniae | 7953 | 700630 | 11351.21 |  |  |
|  | A. baumanii | 4034 | 700630 | 5757.68 |  |  |
|  | S. maltophilia | 747 | 700630 | 1066.18 |  |  |
| 95 | Human herpesvirus 1 | 101501 | 839325 | 120931.70 | F | 60 |
|  | EB virus | 84727 | 839325 | 100946.59 |  |  |
|  | S. aureus | 1172 | 839325 | 1396.36 |  |  |
| 96 | A. baumanii | 51616 | 1026104 | 50302.89 | M | 64 |
|  | P.aeruginosa | 47846 | 1026104 | 46628.80 |  |  |
|  | B. multivorans | 25215 | 1026104 | 24573.53251 |  |  |
|  | S. maltophilia | 15837 | 1026104 | 15434.11 |  |  |
|  | C. parapsilosi | 433 | 1026104 | 421.9845162 |  |  |
|  | Cytomegalovirus | 109 | 1026104 | 106.2270491 |  |  |
|  | K. pneumoniae | 849 | 1026104 | 827.40 |  |  |
| 97 | S. pneumoniae | 5970 | 1564260 | 3816.50 | F | 14 |
|  | S. aureus | 231 | 1564260 | 147.6736604 |  |  |
|  | C. albicans | 89 | 1564260 | 56.89591244 |  |  |
|  | H.influenzae | 1034 | 1564260 | 661.02 |  |  |
